# Disease-associated AIOLOS variants lead to immune deficiency/dysregulation by haploinsufficiency and redefine AIOLOS functional domains

**DOI:** 10.1172/JCI172573

**Published:** 2024-02-01

**Authors:** Hye Sun Kuehn, Inga S. Sakovich, Julie E. Niemela, Agustin A. Gil Silva, Jennifer L. Stoddard, Ekaterina A. Polyakova, Ana Esteve Sole, Svetlana N. Aleshkevich, Tatjana A. Uglova, Mikhail V. Belevtsev, Vladislav R. Vertelko, Tatsiana V. Shman, Aleksandra N. Kupchinskaya, Jolan E. Walter, Thomas A. Fleisher, Luigi D. Notarangelo, Xiao P. Peng, Ottavia M. Delmonte, Svetlana O. Sharapova, Sergio D. Rosenzweig

**Affiliations:** 1 Immunology Service, Department of Laboratory Medicine, Clinical Center, NIH, Bethesda, Maryland, USA.; 2 Research Department, Belarusian Research Center for Pediatric Oncology, Hematology and Immunology, Minsk, Belarus.; 3 Division of Allergy and Immunology, Department of Pediatrics, Morsani College of Medicine, University of South Florida, Tampa, Florida, USA.; 4 Division of Allergy and Immunology, Department of Medicine, Johns Hopkins All Children’s Hospital, St. Petersburg, Florida, USA.; 5 Division of Pediatric Allergy and Immunology, Massachusetts General Hospital for Children, Boston, Massachusetts, USA.; 6 Laboratory of Clinical Immunology and Microbiology, Division of Intramural Research, National Institute of Allergy of Infectious Diseases, NIH, Bethesda, Maryland, USA.; 7 Department of Genetic Medicine, Johns Hopkins University School of Medicine, Baltimore, Maryland, USA.

**Keywords:** Immunology, Adaptive immunity, Molecular genetics, Monogenic diseases

## Abstract

AIOLOS, also known as IKZF3, is a transcription factor that is highly expressed in the lymphoid lineage and is critical for lymphocyte differentiation and development. Here, we report on 9 individuals from 3 unrelated families carrying AIOLOS variants Q402* or E82K, which led to AIOLOS haploinsufficiency through different mechanisms of action. Nonsense mutant Q402* displayed abnormal DNA binding, pericentromeric targeting, posttranscriptional modification, and transcriptome regulation. Structurally, the mutant lacked the AIOLOS zinc finger (ZF) 5–6 dimerization domain, but was still able to homodimerize with WT AIOLOS and negatively regulate DNA binding through ZF1, a previously unrecognized function for this domain. Missense mutant E82K showed overall normal AIOLOS functions; however, by affecting a redefined AIOLOS protein stability domain, it also led to haploinsufficiency. Patients with AIOLOS haploinsufficiency showed hypogammaglobulinemia, recurrent infections, autoimmunity, and allergy, but with incomplete clinical penetrance. Altogether, these data redefine the AIOLOS structure–function relationship and expand the spectrum of AIOLOS-associated diseases.

## Abstract 2

<p>AIOLOS variants Q402* or E82K lead to disease-associated AIOLOS immunodeficiency and immune dysregulation by haploinsufficiency through distinct biochemical mechanisms.</p>

## Graphical abstract

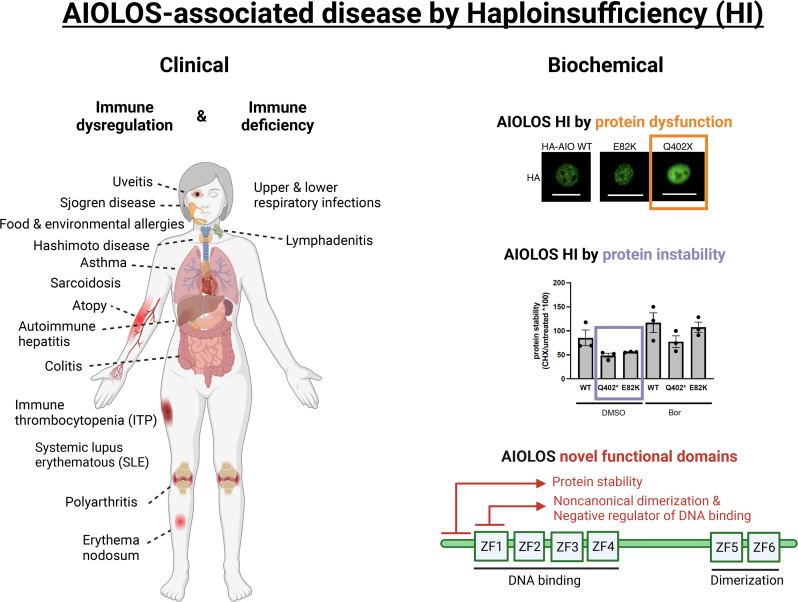

## Introduction

The IKAROS family members consist of 5 zinc-finger (ZF) transcription factors encoded by *IKZF1* to *IKZF5*. IKAROS (*IKZF1*), HELIOS (*IKZF2*), and AIOLOS (*IKZF3*) are highly expressed in the lymphoid lineage, having pivotal roles in lymphocyte differentiation and development (1, 2). Moreover, they also function as tumor suppressors with abnormal alternative splicing and somatic mutations frequently found in B or T cell acute lymphoblastic leukemia (ALL) and/or chronic lymphocytic leukemia (CLL) (3–7). Structurally, all IKAROS family members share 2 well-defined functional domains: an N-terminal DNA-binding domain primarily represented by ZF1 through 4 and a C-terminal dimerization domain encompassing ZF5 through 6 (8, 9).

AIOLOS mutations have also been described in primary immunodeficiency (PID)/inborn errors of immunity (IEI). To date, 2 germline mutations (G159R and N160S) were reported in 2 different families with PID/IEI (10, 11). The G159R mutation is associated with B cell deficiency, recurrent sinopulmonary infections, abnormal T cell differentiation (increased memory phenotype in CD4 T cells), and increased susceptibility to Epstein-Barr virus (EBV) infection and B cell lymphoma (10). In contrast, patients carrying the N160S mutation have no defect in B cell numbers but have abnormal B cell phenotypes with increased numbers of CD21^lo^ B cells, low levels of expression of surface IgM and IgD, impaired CD23 expression, and defects in CD40L signaling. Additionally, abnormalities of the T cell compartment have been also reported, including increased proportions of naive T cells and recent thymic emigrants (RTEs), decreased T follicular helper (Tfh) cells and Th1 population, and impaired CD40 upregulation upon stimulation (11). Characteristically, patients with the AIOLOS N160S mutation present with *Pneumocystis* *jirovecii* pneumonia (PCP) with or without CLL. Despite being only 1 amino acid apart (i.e., G159R and N160S) within the same AIOLOS ZF2 domain, different immunologic and clinical presentations were determined by the different mechanisms of action for the mutations. While G159R exerted a dominant negative (DN) effect on both AIOLOS and IKAROS WT protein’s function (i.e., pericentromeric targeting and DNA binding), N160S showed a DN effect on AIOLOS WT’s function but not IKAROS WT protein’s function.

Here we describe 2 AIOLOS variants in 9 patients from 3 unrelated families manifesting with hypogammaglobulinemia, recurrent respiratory tract infections, and immune dysregulation with incomplete clinical penetrance, somehow resembling clinical manifestations of IKAROS haploinsufficiency (HI) and dimerization defective (DD) mutations (12, 13). Unlike the 2 previously reported AIOLOS mutations affecting ZF2 in the DNA-binding domain and exerting a DN effect on AIOLOS WT and/or IKAROS WT protein, we demonstrate that both mutants herein reported cause AIOLOS-associated immunodeficiency by HI through distinct biochemical mechanisms. This study not only expands the spectrum of AIOLOS-associated diseases in patients with PID/IEI, but also redefines the structure-function relationship of AIOLOS protein.

## Results

### Clinical history

#### Family A.

The index patient (A.II.1) is a 16-year-old Belarusian woman with a history of recurrent upper respiratory tract infections, bilateral otitis media, sinusitis, bronchitis, bilateral submandibular lymphadenitis, and urinary tract infections. Immune thrombocytopenia (ITP) was first diagnosed at age 9 years and recurred at ages 12 and 13 years; each episode showed a partial and unsustained response to steroids and/or intravenous IgG therapy. At age 16 years, her thrombocytopenia became chronic. The patient also had an episode of pityriasis rosea at age 9 years. Based on initial laboratory examinations at the age of 13 years, she was diagnosed with hypogammaglobulinemia based on low IgG levels (< 500 mg/dL), but IgA and IgM levels were within normal limits. Immune phenotyping at the age of 16 years was remarkable for decreased absolute numbers of CD3 and CD4 T cells, RTEs, and B cells, particularly memory B cells (Supplemental Table 1; supplemental material available online with this article; https://doi.org/10.1172/JCI172573DS1). At the time of immune evaluation, the patient was not receiving immunosuppressive medication. Further immunophenotyping studies showed Th1, Th2, Th17, and CD40L expression comparable to healthy donor controls (Supplemental Figure 1A). In vitro studies showed that the patient had normal levels of B cell proliferation and plasmablast differentiation (Supplemental Figure 1B). However, low levels of IgG and IgA production were detected in cultures of the patient’s B cells stimulated with CD40L and IL-21 (Supplemental Figure 1C)

The index patient’s father (A.I.1) had recurrent respiratory infections as a child — not severe, invasive or opportunistic; he did not receive prophylactic antibiotics or IgG replacement therapy — and as an adult he has a history of food allergies. Her younger sister (A.II.2) was diagnosed with chondropathy, which mostly manifested as leg pain, at the age of 9 years, and her mother is healthy.

#### Family B.

B.I.1, the index patient, is 72-year-old American white woman. She had a history of recurrent sinopulmonary infections since young adulthood that worsened in her 50’s, when presenting with multiple ear infections as well as X-ray–proven pneumonias and sinusitis, responsive either to oral or IV antibiotics (no records of proven infectious agents isolated). A chest CT highlighted mild-to-moderate bronchiectases at the pulmonary apexes and bilateral scattered diffuse areas of ground glass nodularities. At age 62, the patient was found to have low serum IgG (572 mg/dL) and IgM (28 mg/dL) levels, as well as impaired response to Pneumovax, which prompted the initiation of IgG replacement therapy (IgGRT) that markedly diminished the frequency and severity of her recurrent infections. Since her 40’s, she has suffered from Hashimoto’s thyroiditis, currently on replacement therapy with levothyroxine, and erythema nodosum that was confirmed by a skin biopsy. At age 52 she was diagnosed with sarcoidosis affecting her lungs and liver; nodules were visualized by CT and MRI imaging, and histopathologically evaluated by tissue biopsies. Assays for angiotensin-converting enzyme [ACE] and antineutrophilic cytoplasmic antibody (ANCA) have been persistently negative, while anti-smooth–muscle autoantibody, known to be associated with autoimmune hepatitis, was found to be mildly positive (1:40, while the control values are less than 1:20). No other autoantibodies were identified — including negative anti-type I–IFN autoantibody testing — that were evaluated due to a persistent Herpes Zoster infection. Bilateral uveitis was diagnosed at age 65 and responded to topical steroids. As systemic immunomodulatory therapy, the patient received short courses of prednisone followed by mycophenolate-mofetil to treat her liver, lung, and cutaneous manifestations of immune dysregulation, which are currently under control.

The index patient’s daughter (B.II.1) is 43-year-old woman and has been diagnosed with systemic lupus erythematosus (SLE) and autoimmune hepatitis since her 20’s. She also suffers from asthma, recurrent sinusitis s/p surgery, and environmental and drug allergies since childhood. The index patient’s son (B.II.2, 38 years old) suffered recurrent ear infections requiring ET tubes, recurrent strep pharyngitis, and 2 episodes of Scarlet fever in childhood. Asthma and environmental allergies were also diagnosed, and he is positive for anti-phospholipid antibodies. He also suffers from stage-3 chronic kidney disease due to posterior urethral valves.

#### Family C.

The index patient (C.II.1) is a 49-year-old white American woman with Hashimoto’s thyroiditis and an atypical Sjogren’s-like sicca syndrome since age 38. Primary features are keratoconjunctivitis sicca and extremely dry mouth leading to difficulty chewing and swallowing, plus polyarthritis. Her serological tests are notable for positive anti-nuclear antibody (ANA) titers (1/320), low positive Ro52, IgM anticardiolipin, and rheumatoid factor, along with negative anti-SSA/SSB autoantibodies. She also suffered from severe atopy since childhood, for which, over many years, she received short courses of oral steroids (maximum dose of 15 mg/day of prednisone), hydroxychloroquine, methotrexate, and omalizumab, all with partial and unsustained responses. Other comorbidities include partial adrenal insufficiency (on physiologic hydrocortisone replacement), orthostatic intolerance, chronic headaches, early menopause, and recurrent subconjunctival hemorrhages. Her infectious history is remarkable for recurrent upper respiratory infections empirically treated with antibiotics with no microorganism isolation; no history of severe, invasive or opportunistic infections was reported. Her lymphocyte phenotyping and serum immunoglobulin evaluations in the last 10 years (while off immunomodulatory medications) only demonstrated slightly reduced B cells and slightly increased IgM levels. The index patient’s mother (C.I.1, 73 years old) was diagnosed with polymyalgia rheumatica, Hashimoto’s thyroiditis, asthma, noninfectious colitis, and atopy; no severe, invasive, or opportunistic infectious were reported. The index patient’s daughter (C.III.1, 13 years old) has a history of food intolerance from age 6 months to 6 years that spontaneously resolved, and recurrent ear effusions/infections in infancy requiring bilateral pressure equalizer (PE) tube placement with complete resolution. No severe, invasive, or opportunistic infections were reported. Immune evaluation, including lymphocyte phenotyping, immunoglobulin levels, and antibody titers to diphtheria and tetanus toxoids were overall appropriate; antipneumococcal antibodies were not protective when evaluated approximately 12 years after completing her pneumococcal conjugate vaccine (PCV) 13 vaccination schedule. C.III.1’s father was also diagnosed with ear effusions/infections in infancy requiring PE tubes, asthma, and environmental allergies. A more extended and summarized data on all patients’ laboratory evaluations are presented in Supplemental Table 1.

### Genetic identification and protein expression of IKZF3/AIOLOS

**Figure 1:**
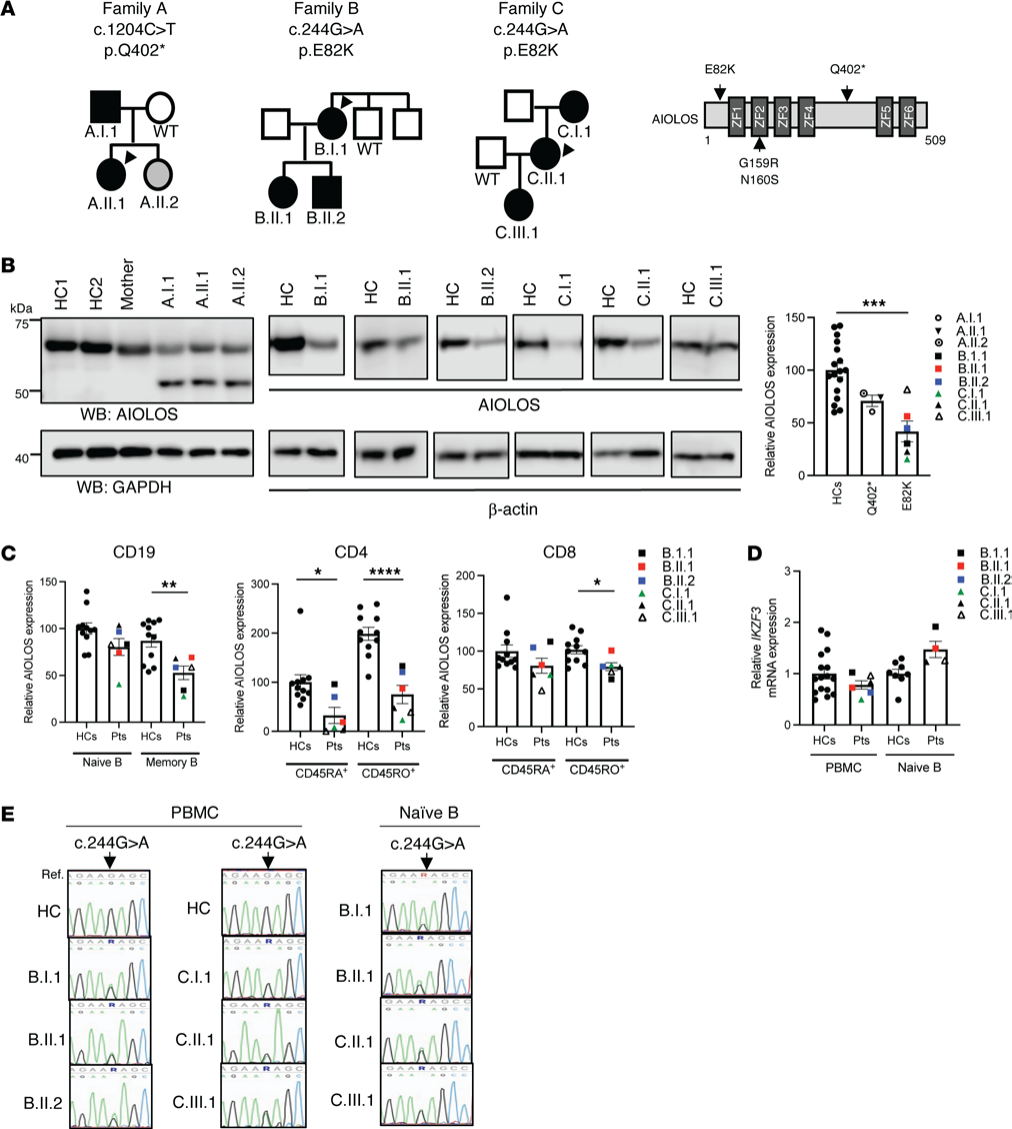
AIOLOS protein and gene expression levels in the patients with heterozygous *IKZF3* variants. (**A**) The pedigrees of the patients with heterozygous *IKZF3* variants (NM_012481). Black and gray colors indicate symptomatic and asymptomatic mutation carriers, respectively. Schematic representation of AIOLOS mutations and ZF domains. Previously reported AIOLOS variants are indicated below the protein. (**B**) AIOLOS protein expression in total PBMCs from the mutation carriers and healthy controls (HC, the age of people in the HC group used in the assay ranges from 29–77). For C.III.1, age matched controls were used. GAPDH or β-actin was used as a loading control. Representative images from 2 experiments are shown. Densitometry analysis of AIOLOS proteins (approximately 60 kDa) normalized by the loading controls. The average value for HCs was set at 100. (**C**) AIOLOS expression was analyzed by flow cytometry in naive and memory subsets from T cells (CD45RA^+^RO^–^ versus CD45RA^–^RO^+^, respectively) or B cells (IgD^+^CD27^–^ versus IgD^–^CD27^+^, respectively). (**D**) The *IKZF3* mRNA levels in PBMCs or naive B cells from healthy controls and indicated patients with the E82K mutation. Relative gene expression levels from PBMCs and naive B cells are shown. Dots represent the average values of technical replicates per sample. (**E**) The chromatograms show the sequence of the heterozygous mutation in cDNA prepared from PBMCs or naive B cells. Data present means ± SEM. Significance was determined by 2-tailed Student’s *t* test, * *P* < 0.05; ** *P* < 0.01, *** *P* < 0.001; **** *P* < 0.0001.

Whole exome sequencing (WES) was performed on the index patients from each of the 3 unrelated families as they were evaluated for genetically undiagnosed PID/IEI and *IKZF3* variants were identified and prioritized. Patient A.II.1 harbors a novel heterozygous nonsense variant in *IKZF3* (Chr17(GRCh37): g.37922369G>A, NM_012481.4(*IKZF3*), c.1204C>T, p.Q402*) (Figure 1A); no other variants in PID/IEI-associated genes were detected. Based on the American College of Medical Genetics and Genomics (ACMG) and the Association for Molecular Pathology (AMP) 2015 guidelines, this variant is classified as of “uncertain significance” (PM2: absent from controls in population databases [gnomAD no frequency]; The CADD GRCh38-v1.6 phred score is 37; PP3: multiple lines of computational evidence support a deleterious effect).

Sanger sequencing of other family members confirmed her father (A.I.1) and younger sister (A.II.2) also carried the same variant. The unrelated index patients B.I.1 and C.II.1 carried the same heterozygous missense variant in *IKZF3* [IKZF3 (NM_012481.5):c.244G>A (p.E82K); chr17-39792853-C-T (hg38)]. This variant (NCBI dbSNP ID rs369340496) is reported in gnomAD Exomes at a frequency of 0.000115; with 29 heterozygous and no homozygous occurrences reported in 251,258 alleles analyzed. The CADD GRCh38-v1.6 phred score is 23.6, and this variant is classified as of “uncertain significance” according to ACMG criteria (PM1). In silico predictions of pathogenicity are mixed: uncertain (SIFT, LIST-S2, MutationTaster, FATHMM-MKL, DANN, EIGEN PC) and benign (MetaLR/SVM/RNN, REVEL, BayesDeladdAF/noAF, EIGEN, FATHMM, FATHMM-XF, LRT, MutationAssessor, MVP, M-CAP) (Supplemental Table 2). Additional WES and Sanger sequencing of B and C family members confirmed that B.II.1 and B.II.2 (daughter and son to B.I.1, respectively), as well as C.I.1 and C.III.1 (mother and daughter to C.II.1, respectively) also carried the same variant. Although no known pathogenic variants were detected in other PID/IEI-associated genes, heterozygous variants of uncertain significance (VUS) were detected in other PID/IEI–associated genes (B.I.1: *CLPB, MTOR*; C.II.1: *AK2, ARHGEF1, TAP2, SKIV2L*) (Supplemental Table 2). However, most of these genes are associated with genetic disorders that are primarily inherited in an autosomal recessive manner and are not clinically consistent with the phenotypes found in our cohort. In particular, segregation analysis of *ARHGEF1* variants showed that all AIOLOS E82K carriers in Family C also shared the 2 ARHGEF1 variants (NM_004706 c.1154G>A p.R385Q and c.842-7G>A). These variants segregated in a cis/monoallelic manner (inherited from the maternal side of the family), were either classified as “likely benign” or “benign” under ACMG criteria, and did not affect protein level expression (data not shown). Additionally, because ARHGEF1 deficiency is reported as an autosomal recessive primary immunologic disorder, the variants detected in the 3 individuals of Family C (but not in Family B) are unlikely to be disease causing. Similarly, all AIOLOS E82K carriers in Family B shared the MTOR variant (NM_004958; c.3452A>G p.Y1151C). When the MTOR pathway was evaluated in B.II.1 by means of AKT (S473) and S6 (S240) phosphorylation upon B cell receptor stimulation, the response was comparable to that of healthy controls (data not shown). While the *ARHGEF1* and *MTOR* variants are unlikely to be disease causing by themselves, the possibility of these or other genes/VUS acting as disease modifiers in AIOLOS HI cannot be completely or formally excluded. Aside from the AIOLOS E82K mutation detected in Family B and C, no other common immune–related rare (*f* < 0.001) coding or splice variants were detected within or across families.

AIOLOS protein expression was evaluated in patients and healthy controls by immunoblotting from PBMCs. All individuals carrying the heterozygous AIOLOS Q402* mutation showed decreased levels of WT protein (approximately 60 kDa) when compared with healthy controls (median, 71%; range, 61%–78%), as well as the expression of the truncated mutant protein at a reduced molecular weight (approximately 50 kDa) (Figure 1B). The patients with the E82K mutation also showed decreased protein expression when compared with healthy controls (median, 42%; range, 16%–82%) (Figure 1B). Next, we evaluated AIOLOS expression in T and B cell subsets. AIOLOS protein was reduced in the E82K patients’ B and T cells, both naive and memory subsets, but significantly more pronounced in memory cells (Figure 1C). These results could likely influence the overall AIOLOS protein expression in individuals with higher proportions of memory cells (e.g., older individuals) when compared to those with higher proportions of naive cells (e.g., younger individuals). To test if the reduced AIOLOS protein expression was due to mRNA decay of the mutant transcript, we tested transcription levels from the patient’s PBMCs and naive B cells. RT-PCR demonstrated that *IKZF3* transcription levels were comparable between the patients and healthy controls (Figure 1D). When the patient with the E82K mutation’s cDNA samples were sequenced, the mutant transcript allele (c.244 G>A) was detected at similar levels of the WT allele, ruling out mRNA decay (Figure 1E).

### Functional evaluation of AIOLOS mutants Q402* and E82K

**Figure 2:**
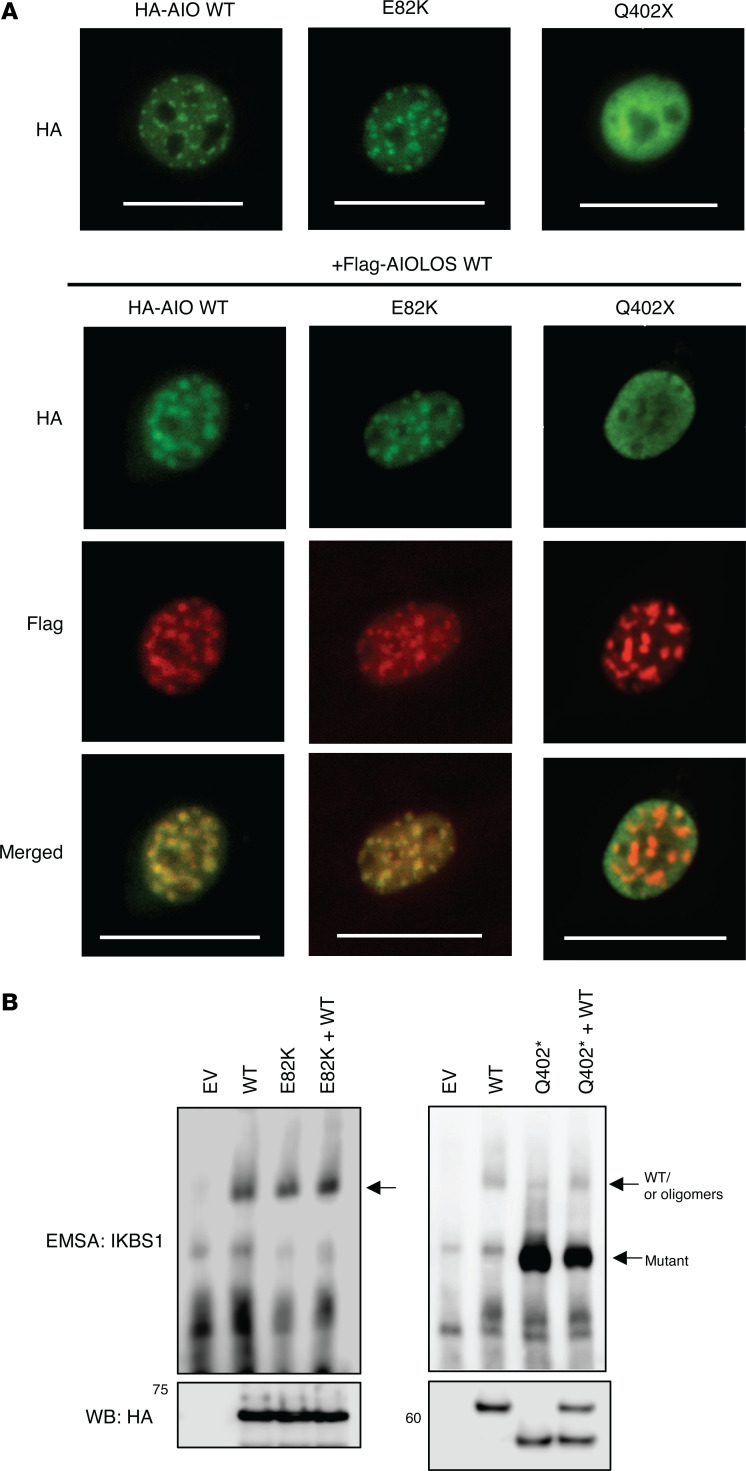
Functional tests for the mutant AIOLOS protein. (**A**) NIH3T3 cells were transfected with HA-tagged WT or the mutant expression vector alone or together with Flag-tagged WT AIOLOS. Cells were labeled with indicated antibodies, followed by Alexa Fluor 488–conjugated and/or Alexa Fluor 568–conjugated secondary antibodies. Cells were visualized using an EVOS (40× objective) fluorescent microscope. Scale bars: 25 μm. (**B**) HEK293T cells were transfected with HA-tagged AIOLOS WT and/or the indicated mutant. Nuclear extracts were prepared and used for the immunoblotting and EMSA assay. Data shown are representative of 3 independent experiments.

**Figure 3:**
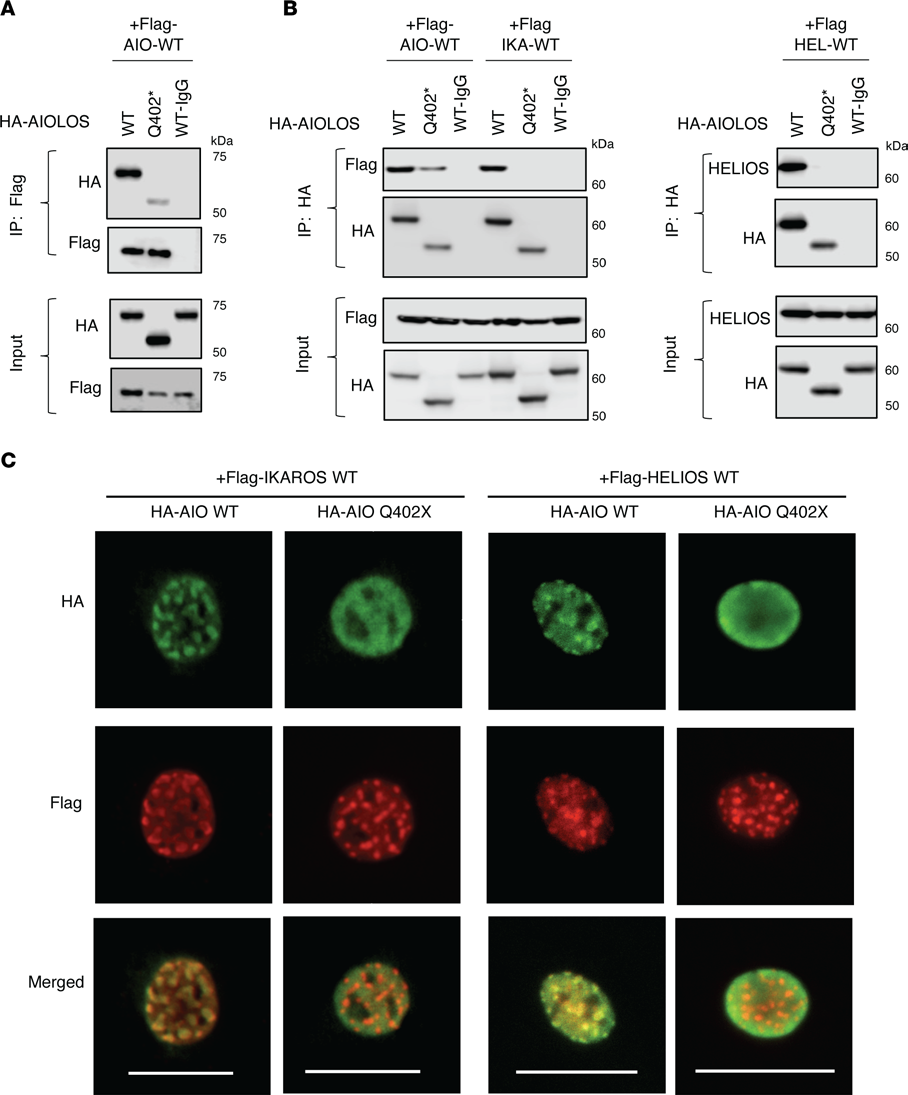
The effect of AIOLOS Q402* on homo- and heterodimerization with IKAROS family members. (**A**) HEK293T cells were transfected with HA-tagged AIOLOS WT or the mutant (Q402*) together with Flag-tagged AIOLOS WT. Immunoprecipitations were performed as indicated in the figure using an anti-rabbit Flag antibody or anti-rabbit IgG control antibody. Western blot data of the IP samples with anti-HA and anti-Flag antibodies are shown. Input controls indicate 5% of the total volumes of the whole cellular lysates used for the IP reaction. (**B**) HEK293T cells were transfected with HA-tagged AIOLOS WT or the mutant together with Flag-tagged AIOLOS WT, IKAROS WT (left panel), or HELIOS WT (right panel). Cell lysates were subjected to immunoprecipitations using anti-rabbit HA antibody or anti-rabbit IgG control. Western blot data of the IP samples with indicated antibodies are shown. (**C**) NIH3T3 cells were transfected with HA-tagged AIOLOS WT or the mutant together with Flag-tagged IKAROS WT (left panel) or HELIOS WT (right panel). Cells were labeled with anti-mouse HA and anti-rabbit Flag antibodies, followed by Alexa Fluor 488-conjugated and/or Alexa Fluor 568-conjugated secondary antibodies, respectively. Cells were visualized using an EVOS (40× objective) fluorescent microscope. Scale bars: 25 μm. Data (**A**–**C**) shown are representative of 3 independent experiments.

**Figure 4:**
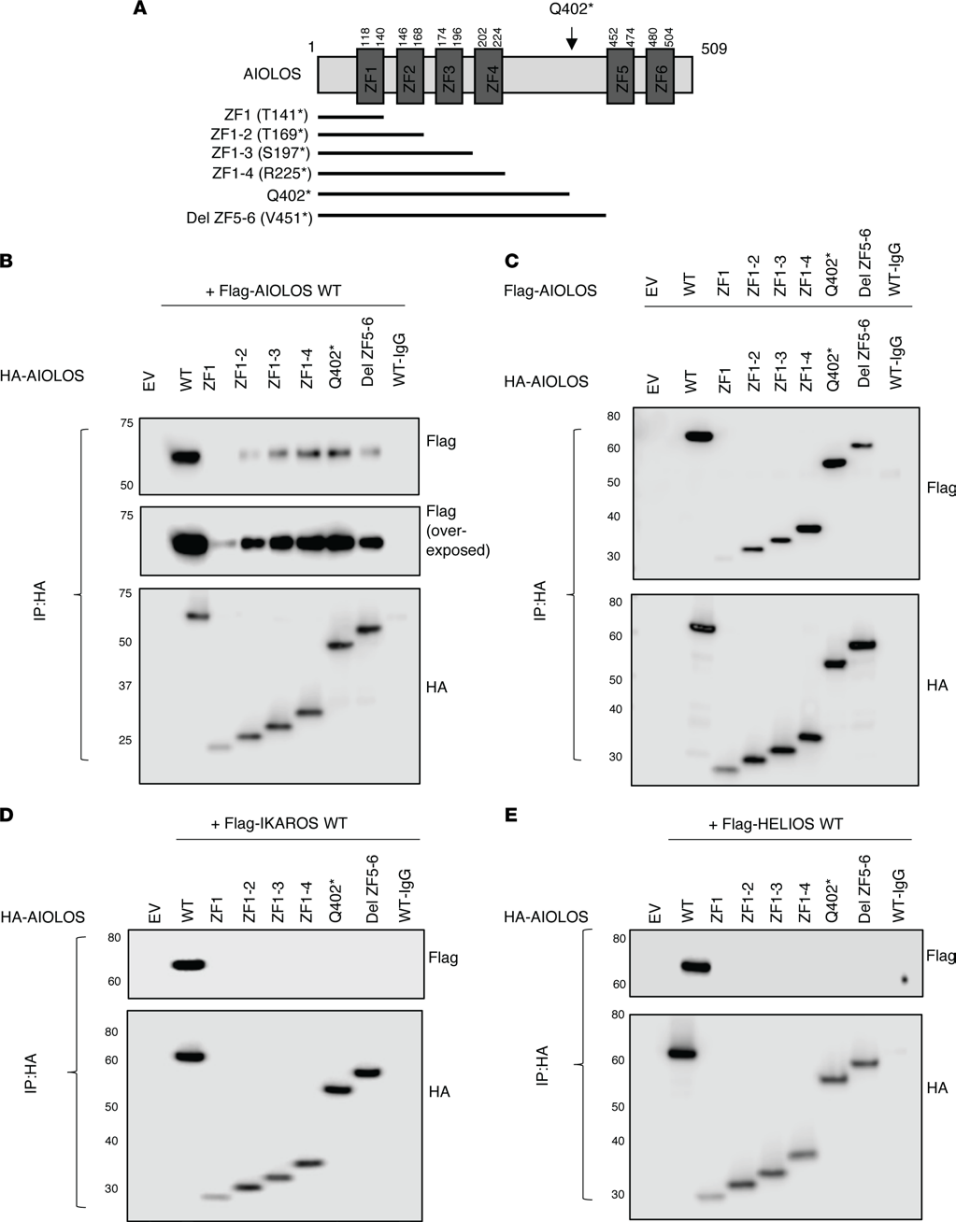
The effect of AIOLOS mutants on homo- or heterodimerization. (**A**) A schematic diagram of AIOLOS mutants is depicted. (**B**–**E**) HEK293T cells were transfected with HA-tagged AIOLOS WT or the indicated mutants together with indicated Flag-tagged IKAROS family members or the AIOLOS mutants. Immunoprecipitations were performed using an anti-rabbit HA antibody or anti-rabbit IgG control antibody. Western blot data of the IP samples with anti-HA and anti-Flag antibodies are shown (please see Supplemental Figure 2, A–D for input controls). Data shown are representative of 3 independent experiments.

**Figure 5:**
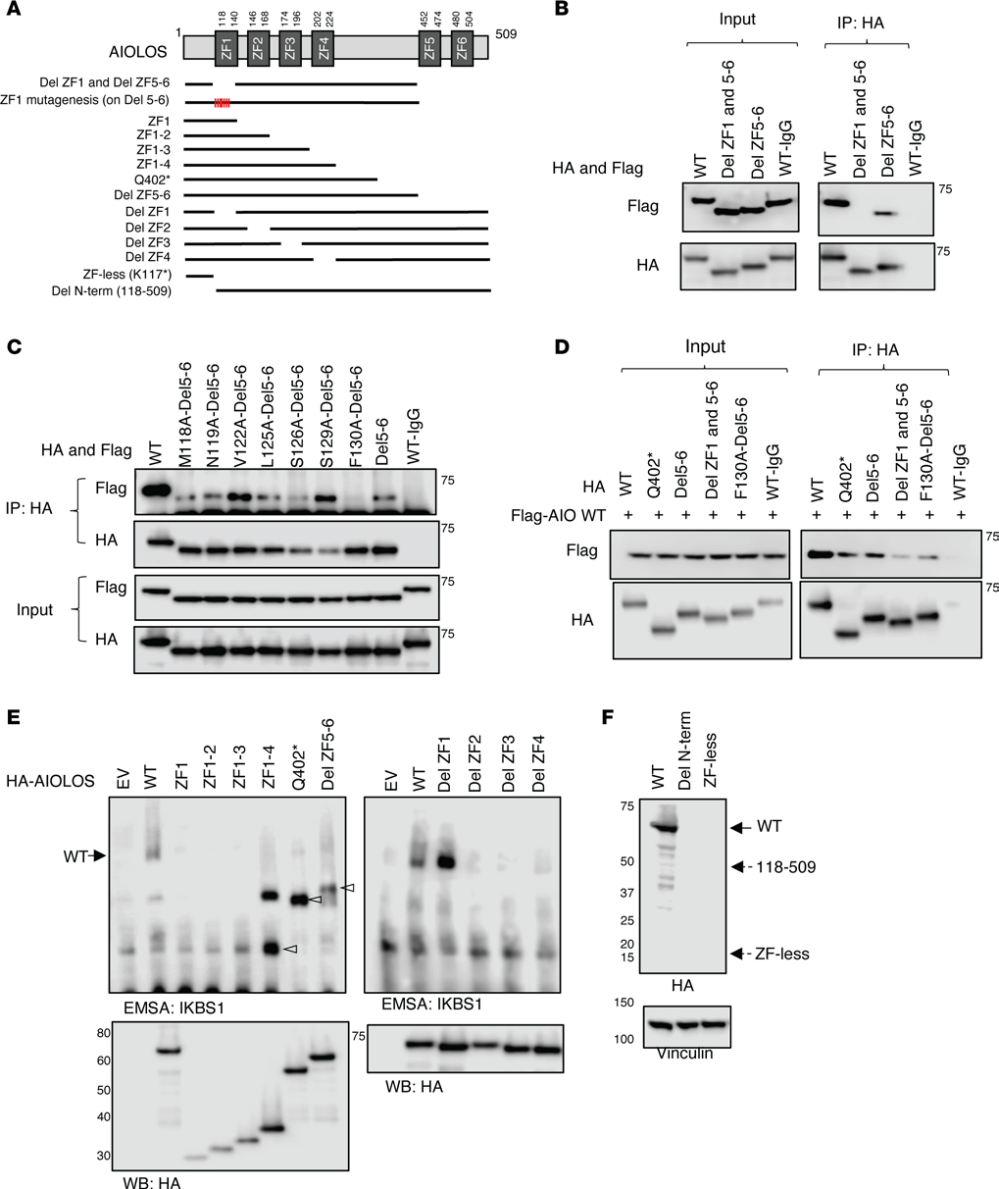
The effect of AIOLOS mutants on the pericentromeric targeting and DNA binding. (**A**) A schematic diagram of AIOLOS mutants is depicted. (**B**–**D**) HEK293T cells were transfected with indicated HA-tagged AIOLOS WT or the indicated mutants together with Flag-tagged AIOLOS WT or the mutants. Immunoprecipitations were performed using an anti-rabbit HA antibody or anti-rabbit IgG control antibody. Western blot data of the IP samples with anti-HA and anti-Flag antibodies are shown. (**E**) HEK293T cells were transfected with HA-tagged AIOLOS WT or the mutants. Nuclear extracts were prepared, and an equal volume of nuclear extracts from each sample was used for the EMSA assay and the protein expression test. (**F**) HEK293T cells were transfected with HA-tagged AIOLOS WT or mutants, and protein expression was tested after 20 to 24 hours transfection. Dotted arrows indicate the expected mutants’ protein size. Vinculin was used as a loading control. Molecular weight markers are shown on the left (kDa). Representative images from 3 independent experiments are shown (**B**–**F**).

Mutant constructs AIOLOS Q402* and AIOLOS E82K were generated to evaluate their functional impact. While AIOLOS E82K did not show any defects in pericentromeric heterochromatin (PC-HC) targeting, AIOLOS Q402* failed to properly localize (Figure 2A). Mutant protein Q402* showed a diffuse staining pattern by itself and did not interfere with the WT protein’s PC-HC targeting in the cotransfection condition, ruling out a DN effect over AIOLOS WT protein (Figure 2A). When DNA binding was tested, mutant E82K was able to bind to its target sequence similar to WT, while mutant Q402* mostly bound at a markedly lower molecular size. This aberrant pattern was highly compatible with monomeric binding and likely due to the failure of dimerization (Figure 2B), as previously observed with IKAROS DD mutations (12). Interestingly, mutant Q402* also displayed a faint binding band of similar molecular size to the WT, suggestive of some residual dimerization or oligomerization capacity. To test whether the Q402* mutant had any impact on the interaction with IKAROS family members, we evaluated its homo- and heterodimerization. Unlike IKAROS DD mutations (e.g., R213* and S427*) (12), which completely failed homo- and heteromerization, AIOLOS Q402* was able to bind to the WT AIOLOS protein, albeit at lower levels than the WT-WT interaction (Figure 3A). In contrast, the mutant completely failed to bind to WT IKAROS and HELIOS proteins, suggesting that the AIOLOS ZF5–6 canonical dimerization domain is required for AIOLOS heterodimerization with IKAROS and HELIOS, but is unlikely to be the only domain involved in AIOLOS homodimerization (Figure 3B). Coexpression of the mutant with WT IKAROS or HELIOS showed similar PC-HC staining patterns as with the WT AIOLOS, demonstrating that the mutant has no DN effect on heterodimeric PC-HC localization (Figure 3C). The reduced but consistent interaction of the Q402* mutant with WT AIOLOS led us to further investigate whether AIOLOS N-terminal domain was involved in AIOLOS-AIOLOS binding. We engineered several AIOLOS mutants (AIO mutants), including N-terminal ZF1 (AIO ZF1; aa 1-140, p.T141*), ZF1 to ZF2 (AIO ZF1-2; aa 1-168, p.T169*), ZF1 to ZF3 (AIO ZF1-3; aa 1-196, p.S197*), ZF1 to ZF4 (AIO ZF1-4; aa 1-224, p.R225*), and ZF1 to ZF4 plus the region upstream of ZF5 and ZF6 (AIO Del ZF5-6; aa 1-450, p.V451*), as shown in Figure 4A. All these mutants expressed comparable protein levels to the WT control, except AIO ZF1, which was slightly reduced. Despite its decreased expression, the presence of AIOLOS ZF1 alone (as in AIO ZF1) was still sufficient to interact not only with the full WT AIOLOS protein but also with AIO ZF1 mutant protein; all mutants completely failed heterodimerization with IKAROS and HELIOS WT proteins (Figure 4, B–E and Supplemental Figure 2, A–D). To test the role of ZF1 in the noncanonical binding, we generated a mutant construct that deleted both ZF1 and the dimerization domain (Del ZF1 and Del ZF5–6). Immunoprecipitation data showed that the noncanonical binding was no longer observed when the ZF1 domain was deleted, suggesting that ZF1 is the major domain mediating this effect (Figure 5, A and B). Since noncanonical dimerization was observed in the AIOLOS but not the IKAROS protein, we hypothesized the discordant amino acids may underlie the difference in their noncanonical dimerization potential. Thus, we introduced alanine mutations at the positions where the AIOLOS amino acids differed from IKAROS protein to further investigate the detailed binding site in ZF1. Immunoprecipitation revealed that phenylalanine at position 130 was critical for noncanonical dimerization as the F130A mutant impaired noncanonical dimerization (Figure 5C and Supplemental Figure 2E). Alongside position F130, additional sites (M118, N119, L125, and S126) also demonstrated a partial effect on noncanonical dimerization (Figure 5C and Supplemental Figure 2E). When we conducted immunoprecipitation assays with AIOLOS WT, we observed that the 2 mutants with completely abolished canonical and noncanonical homodimerization (Del ZF1-Del ZF5-6 and F130A-Del5-6), were still capable of residual binding to AIOLOS WT. However, this interaction occurred at further lower levels when compared with the Del ZF5-6 (Figure 5D and Supplemental Figure 2F), highlighting the role of ZF1 and the F130 site in noncanonical homodimerization. In addition, these results also suggest that an AIOLOS WT allele can sustain minimal and residual homodimerization binding capacity, even with dimerization-incapable mutants. To further examine the DNA binding domain (ZF1–4) on AIOLOS function, we engineered another series of deletion mutants impacting the DNA binding domain and tested AIOLOS function (Figure 5A). EMSA assays showed that AIO ZF1, AIO ZF1–2 and AIO ZF1–3 were unable to bind its target DNA sequence, while mutants AIOLOS Q402* and AIO Del ZF5–6 bound to the DNA sequence primarily as monomers (Figure 5E). Of note, mutant AIO ZF1–4 bound to the target sequence both as monomers and dimers, which is likely dependent on the abnormal homodimerization of the AIOLOS mutants (Figure 4, B and C). Interestingly, mutant construct Del ZF1 (absent ZF1, but the rest of the DNA binding domain [i.e., ZF2-4] remains intact) markedly increased DNA binding compared with the WT control (Figure 5E). In comparison, deletions of ZF2, ZF3, and ZF4 completely failed DNA binding, suggesting that ZF2–4 domains are critical for such function. These findings also suggest that ZF1 likely negatively regulates DNA binding (Figure 5E). PC-HC localization was abnormal in all of the AIOLOS mutant proteins except the mutant with deleted ZF1 (Del ZF1) (Supplemental Figure 3). AIO ZF1, AIO ZF1–2, AIO ZF1–3 and AIO ZF1–4 showed aberrant cytoplasmic accumulation, while AIOLOS mutants Q402* and AIO Del ZF5–6 were detected in the nucleus, yet failed PC-HC targeting (Supplemental Figure 3). These data demonstrate that the DNA binding domain plus at least some of the region between ZF4 and ZF5 is necessary for nuclear localization, but the full canonical dimerization domain is required for proper pericentromeric localization.

To investigate if there was any functional role associated with AIOLOS upstream of ZF1, we generated a mutant construct lacking the first N-terminal 117 amino acids (Del N-term; aa 118–509) while maintaining the full DNA binding domain (ZF1–4) and the dimerization domain (ZF5–6). We found the mutant’s expression level was too low to be detected by immunoblotting (Figure 5F). We also generated a mutant construct that did not contain any ZF domains (ZF-less; K117*), and the mutant was not detected either. The mutant protein (K117*) was probably too short to fold properly, and unable to produce a stable protein. These data suggest that AIOLOS N-terminus integrity is associated with AIOLOS protein stability, which, in part, explains the low AIOLOS protein expression in the patients with E82K mutation mapping to the early N-terminal region. While the AIOLOS protein stability was affected by the E82K mutation, this variant did not affect its homo- and heterodimerization with other IKAROS family members (Supplemental Figure 4A).

**Figure 6:**
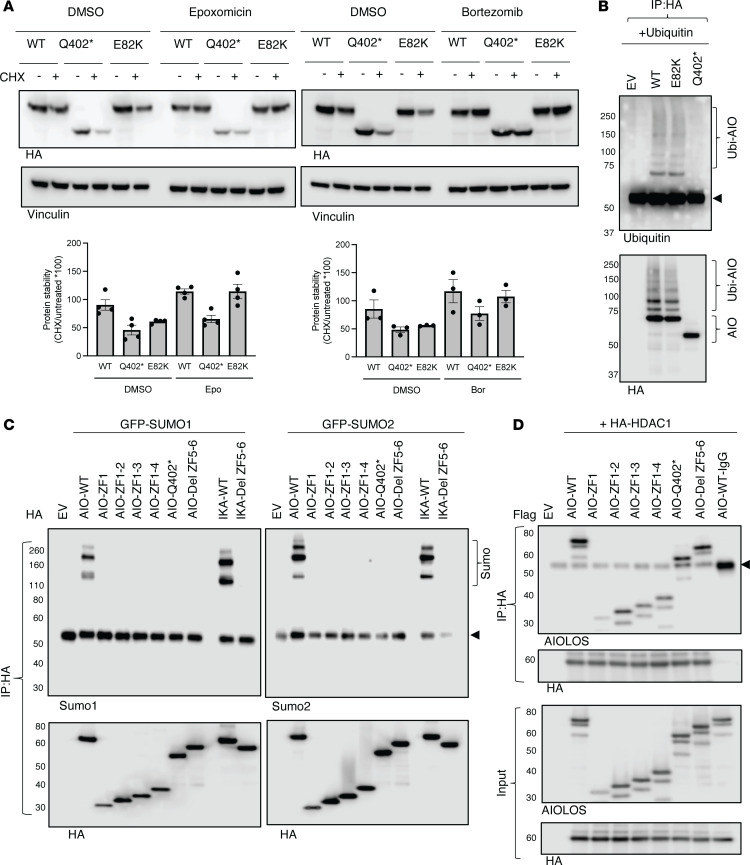
The impact of AIOLOS mutants on protein stability and posttranslational modification. (**A**) HEK293T cells were transfected with indicated vectors. The following day, cells were treated with cycloheximide (CHX, 10 μg/mL) in the presence and absence of epoxomicin (100 nM) or bortezomib (50 nM) for 8 hours. Representative images from 3 to 4 independent experiments are shown. The AIOLOS expression was normalized by the loading control, and the relative AIOLOS protein stability was calculated by dividing the CHX-treated sample by the untreated sample (× 100) for each group. Data indicate mean ± SEM. (**B**) HEK293T cells were cotransfected with HA-AIOLOS and Flag-Ubiquitin. After 48 hours of incubation, cells were treated with Epoxomicin (100 nM) for 3 hours. The protein lysates were prepared and immunoprecipitated under nondenaturing conditions with an anti-rabbit HA antibody. Immunoblot was performed using an anti-ubiquitin antibody or an anti-HA antibody. (**C** and **D**) HEK293T cells were transfected with HA or Flag-tagged AIOLOS WT or the indicated mutant together with GFP-SUMO1/2 or with HA-HDAC1. 24–48 hours after transfection, protein lysates were prepared and subjected to immunoprecipitations. Western blot data of the IP samples with indicated antibodies are shown. Representative images from 3 independent experiments are shown. The arrow indicates the heavy chain of the anti-HA antibody (**B**–**D**).

When AIOLOS mutant protein stability was interrogated after incubation with protein synthesis inhibitor cycloheximide, mutant E82K showed increased degradation compared with the WT control (approximately 40% versus approximately 13% reduction, respectively) (Figure 6A). These results provided extra support for the hypothesis that the reduced AIOLOS protein levels detected in the patients with the E82K mutation were likely the result of mutant protein instability. Of note, mutant Q402*, which already demonstrated aberrant PC-HC localization, DNA binding, and homo- and heterodimerization, also showed increased degradation (approximately 50%) when compared with the WT control. Upon additional evaluation, we demonstrated that the degradation of mutant E82K protein after cycloheximide treatment was prevented by epoxomicin and bortezomib, 2 potent and selective proteasome inhibitors (Figure 6A). While the proteosome inhibitors restored the E82K protein to similar levels as the WT, recovery of Q402* was only partial (Figure 6A). We then investigated whether the mutants had any effect on AIOLOS protein ubiquitination, a mechanism related to proteasome degradation. Interestingly, Q402* exhibited almost absent ubiquitination, whereas E82K demonstrated ubiquitination levels similar to the WT (Figure 6B). These results suggest that the degradation of the mutant E82K is mediated by the ubiquitin-proteasome pathway, while the degradation mechanism of Q402* is likely ubiquitin-independent and therefore different from E82K.

### The effect of the AIOLOS mutants Q402* and E82K on posttranslational modifications and HDAC1 binding

IKAROS has been shown to regulate transcriptional activity through posttranslational modifications, including sumoylation. IKAROS sumoylation attenuates its participation in HDAC-dependent and -independent repression of target genes (14). Our group has shown that the IKAROS dimerization domain is critical for sumoylation as the deletion of ZF5–6 (Y462*) or impaired functional dimerization abrogates this posttranslational modification (12). Sumoylation has also been demonstrated in WT AIOLOS (14). When we tested sumoylation, all AIOLOS mutants except E82K showed abrogated interaction with SUMO1 and SUMO2, suggesting that its canonical dimerization domain (ZF5–6) is necessary for normal sumoylation, and this function could not be supported by the N-terminal noncanonical dimerization domain (Figure 6C and Supplemental Figure 4B).

IKAROS family members have been shown to interact with the NuRD complex and regulate gene transcription. A coimmunoprecipitation assay was performed to test if AIOLOS’ canonical dimerization domain affects its interaction with HDAC1, one of the NuRD complex core members. Unlike IKAROS, where the dimerization domain strongly affects the interaction with HDAC1 (12), AIOLOS interaction with HDAC1 was only minimally affected in mutants that were lacking the ZF5–6 canonical dimerization domain but had a preserved noncanonical dimerization domain (AIO ZF1, AIO ZF1-2, AIO ZF1-3, AIO ZF1-4, Q402*, and AIO Del ZF5-6) (Figure 6D). HDAC1 binding with AIOLOS E82K was also comparable to the WT control (Supplemental Figure 4C).

### Transcriptional dysregulation in the patient’s cells

**Figure 7:**
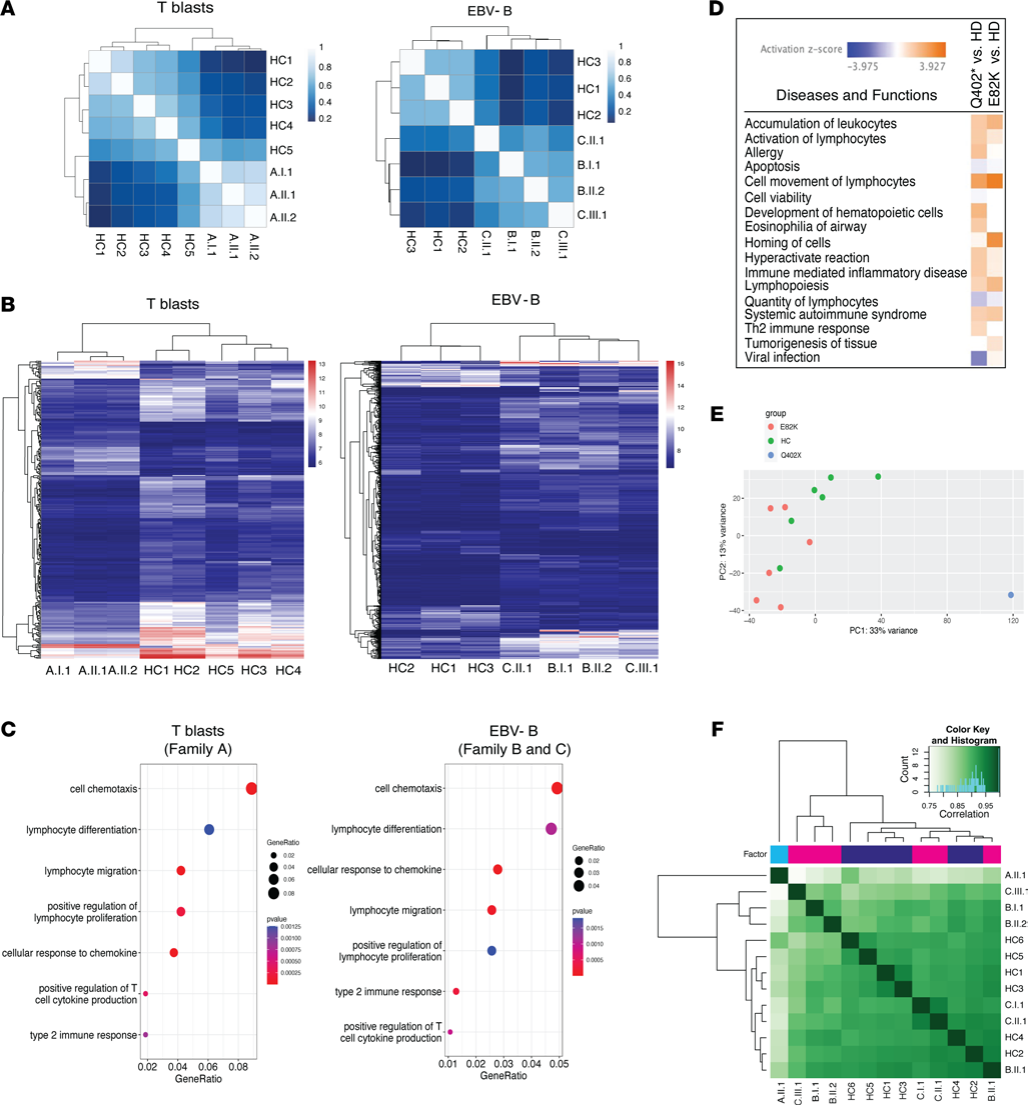
RNA-Seq and ATAC-Seq analyses. (**A**) RNA-Seq was performed on T cell blasts (Q402*) or EBV-transformed B cell lines (E82K). Kendall correlation analyses of the count matrices are shown. The scale bar represents the range of the correlation coefficients (r). (**B**) Heatmap of variance stabilized counts for genes that were determined to be differentially expressed by both the DeSeq2 and CogentDS pipelines. Blue indicates genes with lower expression values and red represents highly expressed genes. (**C**) GO dotplots for selected biological process (BP) that are immune related and shared between T cell blasts (Q402*) and EBV-transformed B-cells (E82K) based on DEGs. DEGs have an adjusted *P* value under 0.01 and absolute log_2_ fold change of greater than 2. The color of the dots indicates the *P* values for each of the terms and the size of the dots depicts the gene ratio. The gene ratio equals the number of differentially expressed genes against the number of genes associated with a GO term in the genome. The *P* value scales indicated in the figure correspond to transformed *P* values across the selected categories. (**D**) IPA Diseases & Functions analysis from the patients with the indicated AIOLOS mutations. (**E** and **F**) Analysis of chromatin accessibility by ATAC-Seq in T cell blasts from healthy controls and the patients with AIOLOS mutations. Principal Component Analysis plot was generated using DeSeq2 (**E**). Correlation heatmap of all chromatin peaks detected by ATAC-Seq was generated by DiffBind (**F**).

To evaluate the transcriptional changes conferred by the AIOLOS Q402* and E82K mutations, we performed RNA-Seq on T cell blasts (Family A) or EBV-transformed B cell lines (Family B and C). Unsupervised clustering correlation analysis revealed that mutation carriers, irrespective of the presence or absence of clinical symptoms, grouped together and separated from healthy controls (Figure 7A). When compared with healthy controls, individuals in Family A carrying variant Q402* showed 284 differentially regulated genes (72 upregulated, 212 downregulated; Figure 7B and Supplemental Table 3). For the patients with E82K mutation, 601 genes were differentially regulated in EBV-transformed B cell line (452 upregulated, 149 downregulated) compared with healthy controls. According to Gene Ontology (GO) analysis, differentially expressed genes in T cell blasts (Q402*) and EBV-transformed B cells (E82K) were enriched in biological processes including lymphocyte differentiation, positive regulation of T cell proliferation, lymphocyte migration, and type 2 immune response (Figure 7C, Supplemental Table 4). Further evaluation using ingenuity pathway analysis (IPA) showed that functions associated with allergy, hypersensitive reaction, immune mediated inflammatory diseases, and systemic autoimmune syndrome were upregulated in both AIOLOS groups (patients with Q402* and E82K mutations), consistent with some of their main phenotypic manifestations, including allergy and immune dysregulation (Figure 7D). To further evaluate whether AIOLOS variants were associated with changes in chromatin accessibility, we performed ATAC-Seq in the patients’ T cell blasts. PCA and heat map analysis showed that, while the HC and the E82K patients mostly clustered together, the largest variance arose from the Q402* versus E82K patients (Figure 7, E and F). A total of 10,555 differentially accessible ATAC-Seq peaks were identified in patient A.II.1 (Q402*), while only 130 were identified in the patients with the E82K mutation. We performed GREAT analysis to identify putative cisregulatory regions that overlapped the differentially accessible ATAC-Seq peaks (adjusted *P* value cutoff under 0.05) and to identify enrichment for the GO Biological Process or Human Phenotype ontologies analyses using nearby genes. Enrichment analyses based on genes around putative cisregulatory regions revealed no enrichment for the GO Biological Process or Human Phenotype ontologies for the patient with the E82K mutation’s group; however, there was significant enrichment in both ontologies for patient A.II.1 (Q402*) (Supplemental Figure 5). For the Q402* ATAC-Seq analysis, 4,770 genes were identified near the putative cisregulatory regions, and 91 were DEGs identified by RNA-Seq (indicated by an asterisk in Supplemental Table 3), suggesting that aberrant binding of AIOLOS Q402* to the cisregulatory regions of these genes may cause abnormal chromatin accessibly, which may alter gene expression. Of note, the patients with the E82K mutation showed minimal changes in chromatin accessibility when compared with the HC. This finding correlates with our overexpression functional studies (e.g., PC-HC localization, DNA binding, sumoylation, and HDAC1 binding) showing that, except for its increased protein instability and the rescue effect by proteasome inhibitors, AIOLOS E82K was more different from Q402* than from AIOLOS WT. In addition, it also reinforces the concept that different pathophysiology mechanisms can underlie AIOLOS HI: i.e., reduced protein function and/or reduced protein stability.

## Discussion

Here, we report 9 individuals from 3 unrelated families harboring heterozygous AIOLOS mutations leading to AIOLOS HI, and in turn, B cell defects, immunodeficiency, and immune dysregulation (Supplemental Table 1 and Supplemental Figure 1C). Through this work we were also able to further refine AIOLOS structural and functional domains.

Recently, germline *IKZF1/*IKARO*S* variants have been shown to be associated with PID/IEI in more than 100 patients (13). Most of the reported variants are either located within the DNA-binding or the dimerization domains, or alter their functions. Clinical and immunological phenotypes vary depending on the mechanisms of action of the IKAROS allelic variants (i.e., HI, DN, DD, and gain-of-function [GOF]) (12, 15–17). Increased susceptibility to infections, T and B cell developmental defects, and a high risk of lymphoid malignancies are shared among 4 allelic variants with markedly variable expressivity and penetrance. Patients with N159S/T mutations that have a DN impact on IKAROS WT function have abnormal T and B cell development and severe infections, including PCP. Most patients carrying HI variants (either no mutant protein expression or mutants with functional DNA binding defects not exerting DN effects) presented with a common variable immunodeficiency (CVID) phenotype and progressive loss of B cells. Patients with DD mutations acting by HI showed a higher risk of hematologic cytopenia and malignancies compared with the other allelic variants. Autoimmunity and immune dysregulation are profound in patients with IKAROS-GOF mutations when compared with the other allelic variants, while allergic diseases and increased plasma cell proliferation were unique to this set of patients. It is of note that IKAROS DN mutations showed complete clinical penetrance, whereas HI, DD, and GOF mutations showed less penetrance, with approximately 25% to 30% of individuals with these allelic variants being asymptomatic.

While only 2 germline AIOLOS mutations have been reported to date in a small number of patients with PID/IEI; AIOLOS-associated diseases also appear to encompass a broad spectrum of clinical and immunological phenotypes. As presented herein, most, but not all, individuals with AIOLOS HI demonstrate abnormalities on their T and B lymphocytes, including low B cell numbers (4 of 9) and/or low switched memory B cells (6 of 9), hypogammaglobulinemia (3 of 9), or inverted CD4/CD8 ratios (2 of 9, caused either by decreased CD4 or increased CD8 cells. It is of note that the phenotype of recurrent bacterial infections and immune dysregulation in AIOLOS HI shows similarities to that in IKAROS HI and DD variants (13) (Supplemental Table 1 and Supplemental Table 5). Unlike the patients carrying the AIOLOS N160S variant with overall low T cell differentiation and CD40L expression (11), the patients with AIOLOS HI had comparable levels of T cell differentiation and CD40L expression compared with healthy controls (Supplemental Figure 1A). B cell proliferation and plasmablast differentiation were also largely within control ranges, yet low in vitro production of IgG and IgA was observed in response to CD40L and IL-21 stimulation in the majority of the patients, suggesting that B cell function under this particular experimental condition was affected in patient B cells (Supplemental Figure 1, B and C). Based on the existing information, we believe the different phenotypes observed between AIOLOS mutations are associated with a gene dosage effect as in the IKAROS-associated diseases (13). While AIOLOS G159R and N160S mutations inhibiting AIOLOS WT — with or without IKAROS WT functions — exhibit complete immunologic and clinical penetrance, AIOLOS Q402* and E82K mutations — acting by haploinsufficiency — presented with incomplete immunological and clinical penetrance at the time this report was prepared.

By investigating these 2 different variants associated with AIOLOS HI, we were able to reformulate the ZF domain functions in AIOLOS (Supplemental Figure 6). AIOLOS ZF1, originally described to be part of its DNA binding domain, appears to actually be a negative regulator of DNA binding and involved in noncanonical homodimerization. Silico modeling by using SPIDER II and PDBePISA predicted that the protein-protein interaction span AIOLOS p.R112 to p.V132, and disruption of the ZF1 tertiary structure would be expected to disrupt homodimerization (data not shown). Our data demonstrate that mutant AIOLOS Q402*, lacking the ZF5–6 canonical dimerization domain, results in impaired heterodimerization with IKAROS and HELIOS, while still capable of residual homodimerization with AIOLOS through ZF1, which acts as a noncanonical dimerization domain. Of note, this previously unrecognized N-terminal noncanonical dimerization function shows lower levels of homodimerization capacity when compared with the AIOLOS WT/WT binding (Figure 3, A and B). Moreover, even though the Q402* mutant can physically homodimerize, it binds to its target DNA sequence (IKBS1) mostly as a monomer, suggesting that the noncanonical N-terminal dimerization domain (within the DNA-binding domain) is not able to substitute for the canonical dimerization domain. Similar to the finding in IKAROS DD mutations, AIOLOS mutant Q402* failed PC-HC localization and sumoylation, demonstrating that both the DNA binding and the canonical dimerization domains are needed for normal AIOLOS functions. Interestingly, when the noncanonical dimerization domain was deleted (Del ZF1), the ability of AIOLOS to bind its target sequence increased (Figure 5E), providing evidence that ZF1 can negatively regulate AIOLOS protein’s DNA binding. It is possible that AIOLOS protein-protein interaction through the noncanonical domain masks the DNA binding domain (ZF2–4) or disrupts the 3-D protein structure, resulting in a reduction in DNA binding. It is of note that a previous study that tested the deletion of ZF1 in IKAROS showed no alteration in its ability to bind target sequences (18). The different function of ZF1 between IKAROS and AIOLOS provides insights that they may play divergent roles recognizing and regulating target genes despite their protein domain and structure similarities.

To further define the biological impact of the N-terminal area, we generated AIOLOS mutants that either excluded all ZF domains (ZF less; p.K117*) or the preZF1 N-terminal region (Del N-term; aa 118-509). In this setting, no mutant proteins were detected in the cell lysates of the HEK293T cells transfected with the ZF less and the Del N-term vectors (Figure 5F), suggesting that protein accumulation, proper folding, or stability was highly dependent on the preZF1 N-terminal region as well as ZF domains. When exploring the mechanism involved in E82K-dependent reduced protein accumulation, we were able to rule out increased mRNA decay of the mutant transcript (Figure 1, D and E). However, protein synthesis interference using cycloheximide showed reduced protein stability of mutant AIOLOS E82K through the ubiquitin-proteasome pathway, likely explaining the decreased amount of AIOLOS protein in the patients carrying this variant (Figure 6A). Even though mutant Q402* also presented decreased protein stability in the cycloheximide chase assay on HEK293T transfected cells, its expression remained stable in the patients’ PBMCs, suggesting that other hematopoietic cell-specific mechanisms may also influence the stability and expression of this mutant. It is also noteworthy that these evaluations and results allowed us to speculate about the potential utility of the FDA-approved proteasome inhibitor Velcade (bortezomib) as a therapeutic molecular target for patients with AIOLOS HI due to the E82K mutation, as in vitro treatment demonstrated the rescue of AIOLOS protein levels. These results are particularly interesting as Velcade has been successfully used for the treatment of immune dysregulation and autoimmune diseases (19-21).

As stated above, AIOLOS HI clinical penetrance was variable. Among the 3 individuals carrying variant Q402*, the index patient presented with a CVID-like phenotype with recurrent infections and severe ITP, her father suffered from food allergies, but her sister was virtually asymptomatic. All carriers of the E82K variant had a combination of infectious disease susceptibility (one with a CVID-like phenotype), autoimmunity, asthma, and allergic diseases. Interestingly, while no asymptomatic carriers were detected in the E82K families studied, 29 likely asymptomatic individuals are reported in gnomAD. This suggests a possible ascertainment bias where asymptomatic E82K carriers are overrepresented in cohorts recruiting for healthy individuals (i.e., gnomAD), while symptomatic individuals are more prevalent when evaluating patients for genetically undiagnosed PID/IEI (i.e., our study). Of note, as per gnomAD design, their data becomes an extremely useful reference sets of allele frequencies to exclude severe pediatric diseases; however, it is possible that some individuals with severe diseases may still be included in their data sets, albeit likely at a frequency equivalent to or lower than that seen in the general population (https://gnomad.broadinstitute.org/about).

Immune dysregulation manifestations, including autoimmunity and allergies, were common features among affected individuals. When Tregs were evaluated among AIOLOS Q402* and E82K mutation carriers, results were overall within normal ranges, as were the expression and upregulation of CD25 upon activation of CD4^+^ T cells (data not shown). On the other hand, when the interaction between overexpressed AIOLOS mutants Q402* and E82K with Treg regulator HELIOS was tested, mutant E82K was able to properly heterodimerize with HELIOS, while Q402* was incapable of doing so (Figure 3, B and C and Supplemental Figure 3A). Although neither of these results are sufficient to rule out or prove a Treg-intrinsic defect, it certainly allows for the speculation that Tregs could be involved in the immune dysregulatory pathophysiology in AIOLOS HI.

In summary, in this work we demonstrated what are, to our knowledge, new insights linked to AIOLOS ZF1 function, including noncanonical dimerization and negative regulation of DNA binding, as well as an important role of the C-terminal dimerization domain for AIOLOS functions and posttranslational modification. Furthermore, we also identified the AIOLOS pre-ZF1 N-terminal region as directly related to protein stability. Finally, we believe that this study defines AIOLOS HI as a clinical entity with incomplete clinical penetrance, expanding the spectrum of AIOLOS-associated diseases in patients with PID/IEI, as well as redefining the mechanistic structure and functions of AIOLOS, a major contributor to immune development and a key member of the IKAROS family of transcription factors.

## Methods

### Cell lines and primary cell culture.

PBMCs and EBV-transformed B cell lines (cell lines generated from the patients’ PBMCs) were cultured in RPMI 1640 supplemented with 10% heat-inactivated FBS, 2 mM L-glutamine, penicillin 100 U/mL, and 100 μg/mL streptomycin (Thermo Fisher Scientific) at 37°C in a humidified 5% CO_2_ incubator. Human embryonic kidney 293T (HEK293T; ATCC, CRL-3216) and NIH3T3 cell lines (ATCC, CRL-1658) were cultured in DMEM supplemented with 10% FBS, L-glutamine, and penicillin/streptomycin (Thermo Fisher Scientific). Cell lines used in this study were frequently tested to be mycoplasma negative.

### Next-generation sequencing.

We used reference genome hg19, and all files necessary for analysis were taken from the platform/resource UCSC Genome Browser (University of California, Santa Cruz, USA). NGS data preprocessing begins with quality assessment and subsequent library purification. Quality assessment was carried out using the fastqc software module (version 0.11.9, https://www.bioinformatics.babraham.ac.uk/projects/fastqc/), which assessed the quality of the resulting library as well as the parameters for subsequent trimming. At the cleaning/filtering/trimming stage, we used the module trimmomatic (version 0.39, http://www.usadellab.org/cms/?page=trimmomatic), which allowed us to correct the read library by removing areas with poor quality, adapter sequences, and primer sequences. The library alignment procedure for the prepared reference was performed using the bwa tool (version 0.7.17, http://bio-bwa.sourceforge.net/). Subsequent processing of the alignment results (filtering, deduplication, and recalibration) was carried out using the samtools (version 1.15.1, http://www.htslib.org/) and picard (version 2.27.4, https://broadinstitute.github.io/picard/) programs.). The GATK modules (version 4.2.3.0, https://gatk.broadinstitute.org/hc/en-us) and ensembl-vep (version 107.0, https://github.com/Ensembl/ensembl-vep), were used to search and annotate/predict the resulting variants. Subsequent filtering and formatting of the annotation results were carried out using the developed software module in the Python language and annovar module (https://annovar.openbioinformatics.org/en/latest/). B.1.1 WES was performed as previously described (11). C.II.1 WES was performed using genomic DNA extracted from peripheral blood mononuclear cells using the Illumina Exome with Enrichment kit and NextSeq 2000 instrument according to the manufacturer’s protocols. Reads were mapped to the UCSC Genome Browser hg38 assembly and variants were called using the Illumina DRAGEN DNA Pipeline. Variants were annotated using ANNOVAR (https://annovar.openbioinformatics.org/en/latest/misc/credit/). The WES data for family A have been deposited in NCBI’s Sequence Read Archive (accession no. PRJNA860330), as well as the WES data for the index patients from family B and C (BioProject ID: PRJNA938105).

### Sanger sequencing.

The *IKZF3* variants that were detected by WES (NM_012481.4 (*IKZF3*), c.244G>A [p.E82K] and c.1204C>T [p.Q402*]) were confirmed by Sanger sequencing on an Applied Biosystems genetic analyzer 3500 (Thermo Fisher Scientific) using the BigDye Terminator v3.1 Cycle Sequencing Kit (Applied Biosystems). Sanger sequencing results were analyzed using a specialized Sequencing Analysis 7.0 software (Applied Biosystems) and BioEdit (Bioedit Ltd). Primers used were as follows: *IKZF3*-forward (for Q402*), 5′-GCTGAGATGTCAAACGGTGC-3′; *IKZF3*-reverese (for Q402*), 5′-TGTTAGGCGAGGTCATTGGT-3′; *IKZF3*-forward (for E82K), 5′-TTCTTGCATTTTCTTCCCTGCAG-3′; *IKZF3*-reverse (for E82K) 5′-AGTATGGCTTCGCTTATGAACCA-3′.

### Bulk RNA-Seq.

Total PBMCs were stimulated with Dynabeads human T-activator CD3/CD28 (Thermo Fisher Scientific) in the presence of IL-2 (10 ng/mL, Peprotech) for 7 days. Fresh IL-2 was added every 2–3 days. RNA from T cell blasts or EBV-transformed B cells was prepared using the Qiagen RNeasy plus mini kit (Qiagen). Illumina libraries were generated using the SMARTer Stranded Total RNA-Seq Kit v3, Pico Input Mammalian Components (Takara). The sequencing libraries were quantified using Bioanalyzer HS reagents and Qubit. Libraries were sequenced on a Illumina HiSeq 2500 instrument using 75 × 75 bp paired-end reads and alignment and mapping analyses were performed by the Takara Cogent NGS Analysis Pipeline v1.5.0 (also known as CogentAP). Differential gene expression analysis and Uniform Manifold Approximation and Projection (UMAP) were performed by the Takara Cogent NGS Discovery Software (CogentDS) and DESeq2 v1.32.0 (Bioconductor version: Release [3.15]). Differentially expressed genes were submitted to IPA (Qiagen). GO enrichment analysis of differentially expressed genes was performed using the GOseq R package (22) and clusterProfiler (23) R packages.

The RNA-Seq data in this publication have been deposited in NCBI’s Gene Expression Omnibus (24) and are accessible through GEO Series accession number GSE207129 (https://www.ncbi.nlm.nih.gov/geo/query/acc.cgi?acc=GSE207129).

### ATAC-Seq.

Total PBMCs were stimulated with Dynabeads human T-activator CD3/CD28 (Thermo Fisher Scientific) in the presence of IL-2 (10 ng/mL, Peprotech) for 10 to 14 days. Fresh IL-2 was added every 2–3 days. ATAC-Seq was performed for T cell blasts for the following study groups: healthy controls (HC, *n* = 6) and patients with AIOLOS E82K (*n* = 6, B.I.1, B.II.1, B.II.2, C.I.1, C.II.1, and C.III.1) and Q402X (*n* = 1, A.II.1). ATAC-Seq was performed using the Active Motif ATAC-Seq Kit following the manufacturer’s protocol and sequenced using the Illumina NextSeq 2000 with 42-bp paired end reads. Using Basepair bioinformatics tools and pipelines (https://www.basepairtech.com/), reads were aligned using Bowtie2 (https://github.com/BenLangmead/bowtie2), and peaks were called with MACS2 (https://github.com/macs3-project/MACS) and annotated using Homer (http://homer.ucsd.edu/homer/motif/). Differential testing, annotation, and visualization were performed using R (v.4.3.1) and the following Bioconductor R packages (https://bioconductor.org/): DESeq2 (v.1.40.2), DiffBind (v.3.10.1), ChIPQC (v.1.36.1), ChIPseeker (v.1.36.0), clusterProfiler (v.4.8.2), and goseq (v.1.52.0). GREAT: Genomic Regions Enrichment of Annotations Tool (v.4.04) (25) was used to identify putative cisregulatory regions in noncoding regions overlapping the ATAC-Seq differentially accessible peaks and to perform enrichment analysis for the GO Biological Process and Human Phenotype ontologies based on genes around the putative cisregulatory elements. The ATAC-Seq data in this publication have been deposited in NCBI’s Gene Expression Omnibus and are accessible through GEO Series accession number GSE244038 (https://www.ncbi.nlm.nih.gov/geo/query/acc.cgi?acc=GSE244038).

### Plasmid preparation.

Human AIOLOS (*IKZF3*) ORF clone (pcDNA3.1/C-(k)DYK-IKZF3, NM_012481) was purchased from GenScript and subcloned into the mammalian expression vector pFlag-CMV2 (Sigma-Aldrich) and pcDNA3-HA using KpnI/XbaI and EcoRI/XhoI sites, respectively. Indicated mutants for the *IKZF3* were generated either by the 2 step PCR method or by the site-directed mutagenesis protocol using AccuPrime Pfx DNA Polymerase (Life technologies), followed by DpnI treatment (New England Biolabs). pCMV6-AC-Myc-DDK-Human IKZF1 (IKAROS, NM_006060) and pEGFP-C1-Sumo1 (NM_003352) used in this study were previously described (12, 15). pCMV3-C-HA-human HDAC1 (NM_004964.2) and pCMV3-GFPSpark-Sumo2 (BC016775) were purchased from Sino Biological. pCMV6-Entry-IKZF2 (HELIOS, NM_016260) was purchased from Origene. pcDNA3.1/C-(k)DYK-Ubiquitin B was purchased from GenScript (UBB, NM_001281720). The detailed primer sequence information is presented in Supplemental Table 6.

### Immunoblotting.

Total PBMCs (0.5 million per sample) were washed with PBS once and lysed in the lysis buffer (50mM Tris pH 7.4 [Invitrogen], 150mM NaCl [Quality Biological], 2mM EDTA [Corning], 0.5% Triton X-100 [Sigma-Aldrich] and 0.5% NP40 [Sigma-Aldrich] and protease and phosphatase inhibitor cocktail [Sigma-Aldrich]). Cell lysates were prepared and separated by NuPAGE Novex 4–12% Bis-Tris Protein Gels (Life Technology) and transferred to nitrocellulose membranes using a Trans-Blot Turbo Transfer system (Bio-Rad). The membranes were incubated with anti-AIOLOS (Cell signaling technology, Cat. 15103S) and anti-GAPDH (Cell signaling technology, Cat. 2118S) antibodies or β-actin (Santa Cruz Biotechnology, Cat. sc-47778), followed by horseradish peroxidase-conjugated second antibodies (Jackson ImmunoResearch, nos. 115-035-003 and 111-135-003) and the target proteins were developed by use of SuperSignal West Dura Extended Duration Substrate (Thermo Fisher Scientific). The images were acquired and analyzed with C-Digit scanner using Image Studio Software (Li-Cor).

### Cycloheximide chase assay.

HEK293T cells were transfected with HA-tagged WT, Q402* or E82K using Effectene (QIAGEN) according to the manufacturer’s instructions. After 16 to 18 hours of incubation, cells were treated with Cycloheximide (10 μg/mL, Sigma-Aldrich) alone or together with Epoxomicin (100 nM, Sigma-Aldrich) or Bortezomib (50 nM, Selleckchem) for additional 8 hours. Cells were lysed and subjected to the immunoblotting using anti-HA (Cell signaling technology, Cat. 2999S) and anti-Vinculin (Santa Cruz Biotechnology, Cat. sc-73614).

### Gene expression.

Total RNA was isolated from total PBMCs with the RNeasy plus mini Kit (QIAGEN). The RNA was reverse transcribed using QuantiTect Reverse Transcription Kit (QIAGEN). The gene expression was analyzed by real-time PCR using Taqman probes (Applied Biosystems, *IKZF3* (Hs00232635) and *GAPDH* (4326317E)) and StepOne Plus real time PCR (Applied Biosystems) according to manufacturer’s instructions. Triplicate reactions of each sample were performed for 40 cycles using TaqMan gene expression master mix (Applied Biosystems). GAPDH was used as the endogenous control. The relative quantification of *IKZF3* was calculated with the comparative Ct method. For each sample, the threshold cycle (Ct) was determined, and the relative fold expression was calculated as follows: ΔCt = Ct of *IKZF3* − Ct of *GAPDH*. ΔΔCt = ΔCt of patient samples − ΔCt of healthy donor controls. The relative fold expression was calculated using the equation 2^–ΔΔCt^.

### Immunoprecipitation.

HEK293T cells were cotransfected with plasmids using effectene transfection reagent (Qiagen) according to the manufacturer’s instruction. Twenty-four to 72 hours after transfection, cell lysates were prepared in the lysis buffer (50mM Tris [pH 7.4], 150mM NaCl, 2mM EDTA, 0.5% Triton X-100, and protease and phosphatase inhibitor cocktail [Sigma-Aldrich]). Total protein lysates (0.5–1 mg) were incubated with a rabbit anti-Flag antibody (Cell signaling technology, Cat.14793S), rabbit anti-HA antibody (Cell signaling technology, Cat. 3724S), or rabbit IgG (Cell signaling technology, Cat. 2729S) together with 40 to 50 μL Protein A/G-agarose beads (Pierce) for 4 to 5 hours at 4°C on a rotating wheel. Beads were washed 3 times with lysis buffer and the IP and total lysate samples were subjected to Western blotting with indicated antibodies (anti-HA [Cell signaling technology, Cat. 2999S], anti-Flag [Cell signaling technology, Cat. 8146S], anti-Helios [Cell signaling technology, Cat. 42427S], and anti-AIOLOS [Cell signaling technology, Cat. 15103S]). Images were analyzed with Image Studio Software (Li-Cor).

For the Sumoylation and Ubiquitination assay, cell lysates were prepared in lysis buffer (50mM Tris pH 7.4, 150mM NaCl, 2mM EDTA, 0.5% NP40, 1% SDS, and 20mM N-ethylmaleimide (Sigma-Aldrich) and protease and phosphatase inhibitor cocktail [Sigma-Aldrich]). For the Ubiquitination assay, cells were pretreated with Epoxomicin (100 nM, Sigma-Aldrich) for 3 hours. The protein lysates were passed through QIAshredder (Qiagen) to reduce viscosity. The protein lysates were subjected to immunoprecipitation using rabbit anti-HA antibody. The IP and total lysate samples were subjected to Western blotting with anti-Sumo1 (Abcam, Cat. ab32058), anti-Sumo2 (Sino Biological, Cat. 101083-T38), anti-Ubiquitin (Cell signaling technology, Cat. 58395S), or anti-HA (HRP Conjugated; Cell signaling technology, Cat. 2999S). Images were analyzed with Image Studio Software (Li-Cor).

### PC-HC localization.

NIH3T3 cells grown on cover slips in 6 well plates were transfected with indicated plasmids using Effectene (Qiagen) or Nucleofector kit R (Amaxa, program A-24) according to the manufacturer’s instructions. Cells were washed twice with PBS, fixed for 10 minutes in 4% paraformaldehyde, permeabilized for 15 minutes in 0.1% Triton X-100 in PBS at room temperature, and blocked for 30 minutes in blocking buffer (PBS with 10% FBS and 0.1% Triton X-100). Cells were incubated for 2 hours with an anti-HA antibody (Cell signaling technology, Cat. 2367S or 3724S, or Biolegend Cat. 901501), and/or anti-Flag antibody (Cell signaling technology, Cat. 14793S), followed by Alexa Fluor 488 (Thermo Fisher Scientific, Cat. A11001 or A11070) and/or Alexa Fluor 568 -conjugated secondary antibodies (Thermo Fisher Scientific, Ca. A21069) for 1 hour. Cells were washed with PBS 2 to 3 times in between each step. Cells were stained with DAPI (1 μg/mL, Cell signaling technology, Cat. 4083S) for 5 minutes, washed, mounted on slides using VECTASHIELD Mounting Medium (Vector Laboratories), and visualized using an EVOS M5000 cell imaging system (40× objective, Thermo Fisher scientific).

### Lightshift chemiluminescent EMSA.

HEK293T cells were transfected with vectors expressing AIOLOS WT or mutants using Effectene (Qiagen) according to the manufacturer’s instruction. After 48–72 hours’ incubation, nuclear extracts were prepared using NE-PER nuclear and cytoplasmic extraction kit (Thermo Fisher Scientific). Protein expressions from nuclear extracts were tested and the nuclear extracts were subjected to gel mobility shift assays by using LightShift Chemiluminescent EMSA kit (Thermo Fisher Scientific) according to the manufacturer’s instruction. The IKBS1 probe was used for EMSA assays. 5′-BIOTIN-TCAGCTTTTGGGAATACCCTGTCA; reverse: 5′-BIOTIN-TGACAGGGTATTCCCAAAAGCTGA.

### Evaluation of cell proliferation.

Total PBMCs were incubated with CellTrace violet (1 μM; Invitrogen) for 20 minutes at 37°C in a humidified 5% CO_2_ incubator. Cells were washed with complete RPMI medium 2 times and stimulated with CD40L (100 ng/mL, Enzo) together with IL-21 (50 ng/mL, Peprotech) or anti-IgM (10 μg/ml, Jackson Labs) together with CpG (500 nM, Enzo) for B cell proliferation. After incubation for 4 to 5 days, cells were stained with fluorochrome-conjugated CD19, CD27, and CD38 (BD Biosciences, clone HIB19, M-T271, and HIT2, respectively) for 30 minutes at 4°C in the dark. Cells were washed with PBS twice and acquired and analyzed by flow cytometry (Becton Dickinson FACSCanto II) and FlowJo software (FlowJo 10.7.1, TreeStar).

### Immunoglobulin secretion.

The supernatants of CD40L and IL-21 stimulated samples from the proliferation assay were saved, and immunoglobulin production was measured from the culture supernatant by ELISA kits (Abcam; Cat. ab195215, ab196263, and ab137982, Thermo fisher; 88-50600-86 and 88-50620-86) according to the manufacturer’s instructions.

### Measurement of Th1, Th2, and Th17 populations and CD40L expression.

Total PBMCs were stimulated with PMA (100 ng/mL, Sigma-Aldrich) and ionomycin (1 μM, Invitrogen) for 5 to 6 hours in the presence of brefeldin A (10 μg/mL, Sigma-Aldrich). The stimulated cells were stained with CD3 and CD4 fluorochrome-conjugated antibodies for 30 minutes and then washed, fixed, and permeabilized with the BD cytofix/cytoperm kit. Cells were stained with the indicated antibodies (anti–IFN-γ [Biolegend 506507, clone B27], anti–IL-4 [BD 561595, clone 8D4-8], anti–IL-17A [BD 560491, clone N49-653]).

For CD40L expression, total PBMCs were stimulated with PMA (100 ng/mL) and ionomycin (1 μM) for 22 to 24 hours. Cells were stained with CD3 and CD8 fluorochrome–conjugated antibodies together with an anti-CD40L antibody [BD 555700, clone TRAP1] and analyzed by flow cytometry. Cells were acquired and analyzed by flow cytometry (Becton Dickinson FACSCanto II) and FlowJo software (FlowJo 10.7.1, TreeStar), respectively.

### AIOLOS protein expression by flow cytometry.

Total PBMCs cells were stained with Pacific Blue anti-CD3 (BD 558117, clone UCHT1), PerCP-anti-CD4 (BD 566924, clone SK3), APC-Cy7-anti-CD8 (BD 557834, clone SK1), FITC-anti-CD45RA (BD 555488, clone HI100), and PE-anti-CD45RO (BD 555493, clone UCHL1) antibodies or APC-Cy7-anti-CD19 (BioLegend 302218, clone HIB19), Pacific Blue-anti-CD27 (BioLegend 356414, clone M-T271), FITC-anti-IgD (Life Technology H15501), and PerCP-anti-CD3 (BioLegend 300326, clone HIT3a). After 30 minutes of incubation, cells were washed, fixed, and permeabilized using the Foxp3/transcription factor staining buffer set (Thermo Fisher Scientific) according to the manufacturer’s instruction. Cells were then intracellularly stained with an Alexa Fluor 647-anti-AIOLOS antibody (BD 565215, clone S50-895) for an hour. Cells were acquired and analyzed by flow cytometry (Becton Dickinson FACSCanto II) and FlowJo software (FlowJo 10.8.2, TreeStar), respectively.

### Statistics.

When indicated, data were analyzed using 2-tailed Student *t* test utilizing the GraphPad Prism software (GraphPad, version 9.5.0). The differences were considered significant when *P* < 0.05.

### Study approval.

All patients or their guardians provided written informed consent in accordance with the Declaration of Helsinki under IRB-approved protocols of the National Institute of Allergy and Infectious Diseases, NIH, Bethesda, Maryland, USA. Blood from healthy donors was obtained under IRB-approved protocols of the NIH Clinical Center, NIH.

### Data availability.

All data needed to evaluate the conclusions in the paper are present in the paper and/or the Supplementary Materials or have been placed in a publicly available repository through NCBI’s Sequence Read Archive (accession no. PRJNA860330 and PRJNA938105) and NCBI’s Gene Expression Omnibus and are accessible through GEO Series accession numbers GSE207129, GSE242931, and GSE244038. Values for all data points shown in graphs are provided in the Supplemental Supporting Data Values file.

## Author contributions

HSK and SDR designed the project and wrote the manuscript. SOS identified the family and was involved in collecting biological and clinical data from the patients. HSK performed experiments, analyzed data, and prepared figures. JEN and JLS performed whole exome sequencing, RNA-Seq, ATAC-Seq, and analyzed data. ISS and VRV performed Exome analysis. AAG and AES performed experiments. EAP performed Sanger sequencing analysis, and ISS, MVB, TVS, and ANK performed immunological evaluation. SNA, XPP, OMD, and TAU were involved in collecting and analyzing clinical data and family history. JEW, LDN, and TAF contributed to clinical insight and scientific discussion.

## Supplementary Material

Supplemental data

Supplemental table 2

Supplemental table 3

Supplemental table 4

Supporting data values

## Version 1 11/28/2023

In-Press Preview

## Version 2 02/01/2024

Electronic publication
