# Supplementary material for: Disease-associated AIOLOS variants lead to immune deficiency/dysregulation by haploinsufficiency and redefine AIOLOS functional domains
Source: J Clin Invest. 2024 Feb 1;134(3):e172573. doi: 10.1172/JCI172573 (PMC10836806; doi:10.1172/JCI172573)
Supplement: Supplemental data [file jci-134-172573-s087.pdf]

**Supplementary Data**

Supplemental Figure 1. T cell phenotypes and B cell functions in AIOLOS haploinsufficiency patients

Supplemental Figure 2. Input control data for Figure 4B-E and quantification of immunoprecipitation data for Figure 5C and D

Supplemental Figure 3. Pericentromeric targeting for the mutant AIOLOS proteins

Supplemental Figure 4. The impact of AIOLOS E82K on homo-heterodimerization, sumoylation, and HDAC1 interaction

Supplemental Figure 5. Enrichment analysis for the Human phenotype ontologies and GO Biological Process for patient A.II.1.

Supplemental Figure 6. Summary of AIOLOS mutations' results

Supplemental Table 1. Laboratory results of immunological tests in AIOLOS haploinsufficiency patients

Supplemental Table 5. Immunological and clinical phenotypes of germline heterozygous *IKZF3* mutations in AIOLOS-associated diseases

Supplemental Table 6. Primer sequences used to generate AIOLOS mutant constructs for this study

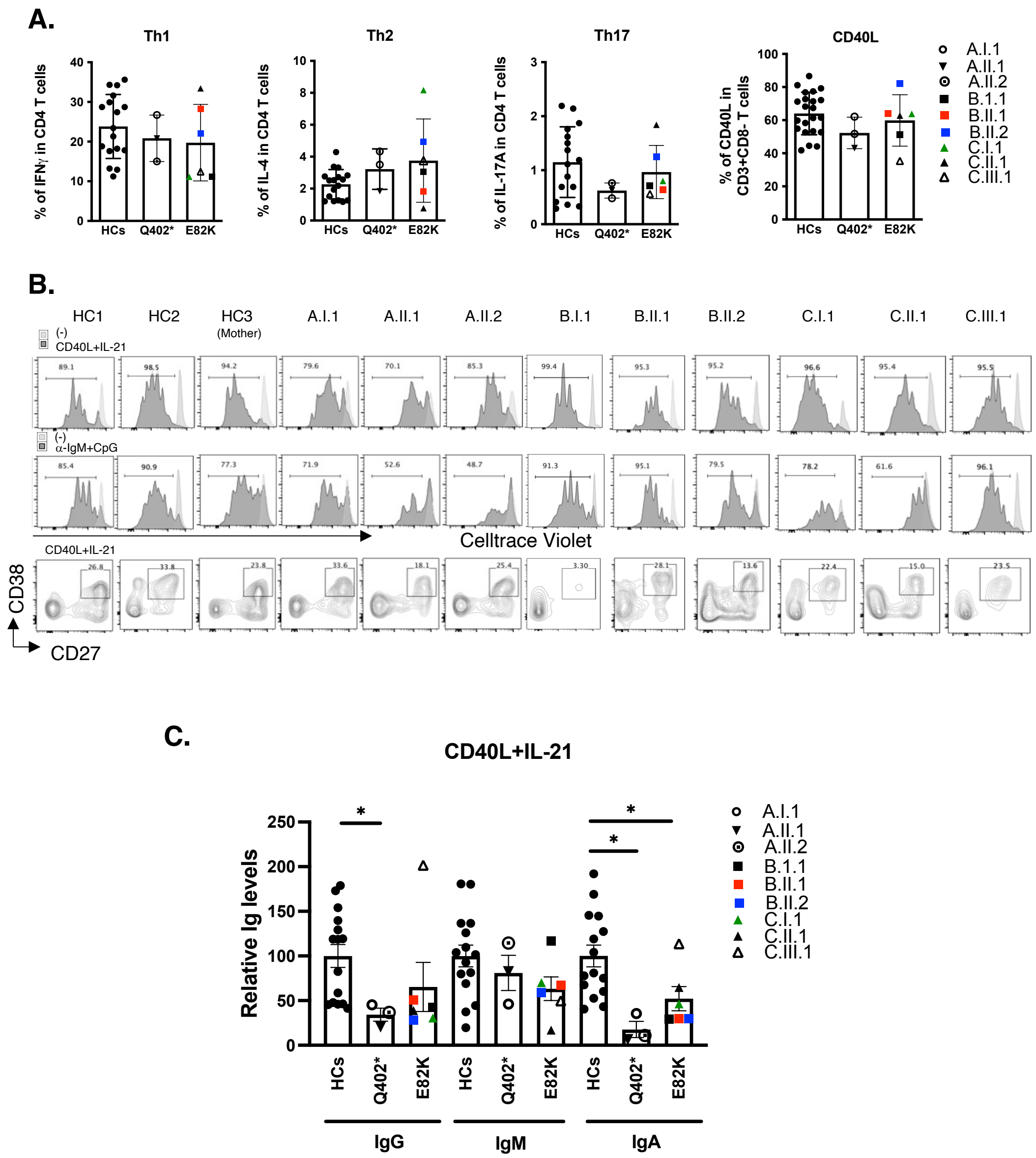


**Supplemental Figure 1. T cell phenotypes and B cell functions in AIOLOS haploinsufficiency patients**

(A) The percentages of IFNγ (Th1), IL-4 (Th2), and IL-17A (Th17) from CD3+CD4+ T cells were shown. Data are mean + SD from healthy normal controls and the patients with indicated mutations. CD40L expression on CD3+CD8- (mostly CD4 T) cells was measured after PMA and ionomycin stimulation. (B) CellTrace Violet stained PBMCs were stimulated with indicated agonists. After 4-5 days stimulation, cells were acquired and analyzed by flow cytometry and FlowJo software, respectively. The numbers indicate the frequency of proliferating B cells. CD40L and IL-21 stimulated B cells were gated on CD27+ and CD38+ for the plasmablast differentiation. Data shown are representative of two experiments. HC3 is the mother of Family A. (C) The supernatants of CD40L and IL-21 stimulated samples from Supplemental Figure 1B were saved, and IgG, IgM, and IgA production was measured by ELISA kits. Data are mean + SEM from healthy normal controls and the individuals with the indicated mutations. The average value for healthy controls was set at 100 and relative Ig values were calculated. Significance was determined by two-tailed Student *t* test, * $p < 0.05$. No patients were on immunosuppressive therapies at the time of Ig evaluation, except for B.I.1 who was on maintenance mycophenolate mofetil therapy.

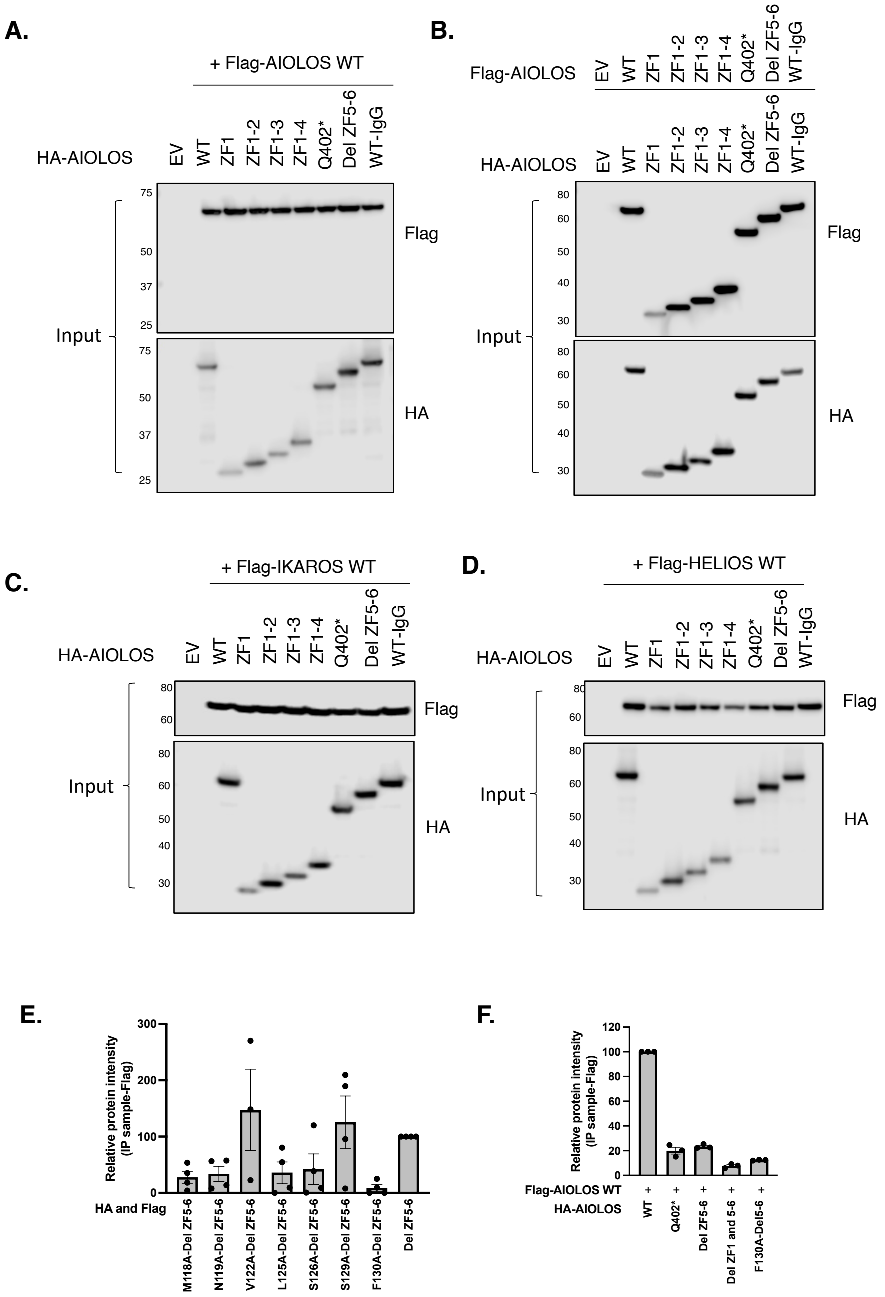


**Supplemental Figure 2. Input control data for Figure 4B-E and quantification of immunoprecipitation data for Figure 5C and D**

(**A-D**) HEK293T cells were transfected with HA-tagged AIOLOS WT or the mutant together with indicated Flag-tagged AIOLOS, IKAROS, HELIOS WT or the AIOLOS mutants. Immunoprecipitations were performed using an anti-rabbit HA antibody or anti-rabbit IgG control antibody. Input controls indicate 5% of the total volumes of the whole cellular lysates used for IP reaction. Western blot data for the input controls are shown. Representative images from three independent experiments are shown. (**E**) The densitometry data for Figure 5C. The AIOLOS protein intensity was normalized to the value of Del ZF5-6 (V451*). Data present means ± SEM. (**F**) The densitometry data for Figure 5D. The AIOLOS protein intensity was normalized to the value of AIOLOS WT. Data present means ± SEM.

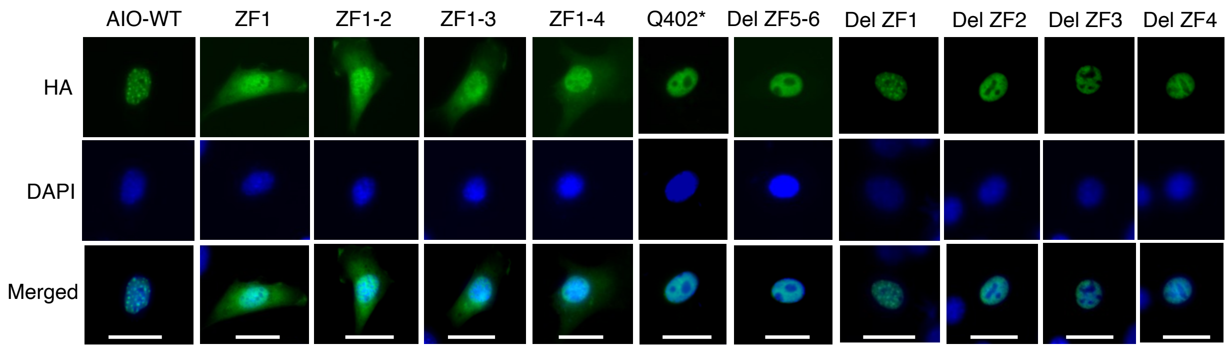


**Supplemental Figure 3. Pericentromeric targeting for the mutant AIOLOS proteins**
NIH3T3 cells were transfected with HA-tagged AIOLOS WT or the mutants as described in the figure. The transfected cells were stained with an anti-HA antibody, followed by an Alexa 488-conjugated secondary antibody (green) and DAPI (blue), and then visualized using an EVOS (40X objective) fluorescent microscope. The scale bars indicate 25 µm. Representative images from three independent experiments are shown

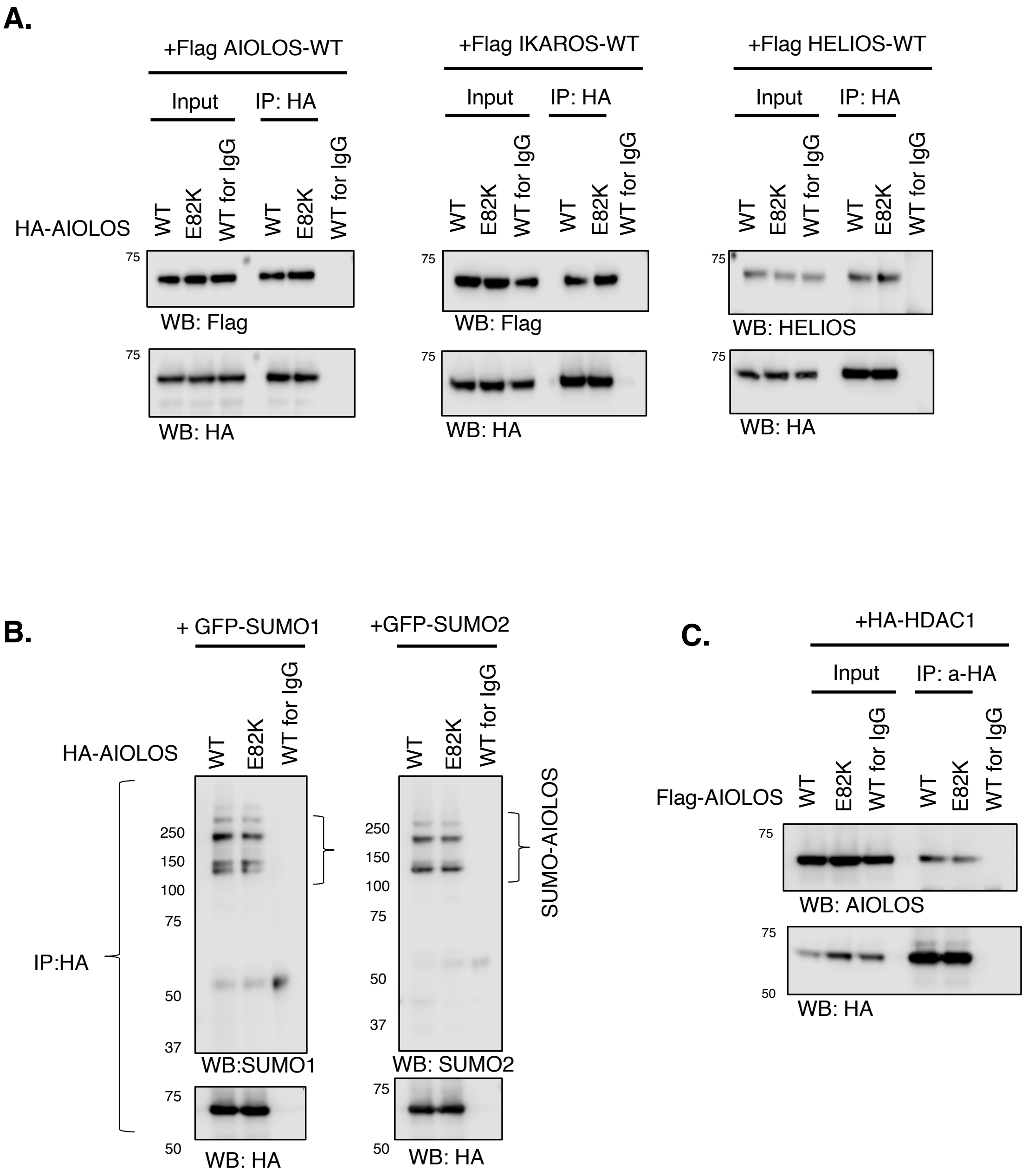


**Supplemental Figure 4. The impact of AIOLOS E82K on homo-heterodimerization, sumoylation, and HDAC1 interaction**

(**A**) HEK293T cells were transfected with HA-tagged AIOLOS WT or the mutant together with indicated Flag-tagged IKAROS family members. Immunoprecipitations were performed using an anti-rabbit HA antibody or anti-rabbit IgG control antibody. Western blot data of the IP samples with indicated antibodies are shown. (**B** and **C**) 293T cells were transfected with the AIOLOS WT or the mutant with GFP-SUMO1/2 or with HA-HDAC1. 24-48 hours after transfection, protein lysates were prepared and subjected to immunoprecipitations, followed by immunoblotting with indicated antibodies. Representative images from three independent experiments are shown.

A.

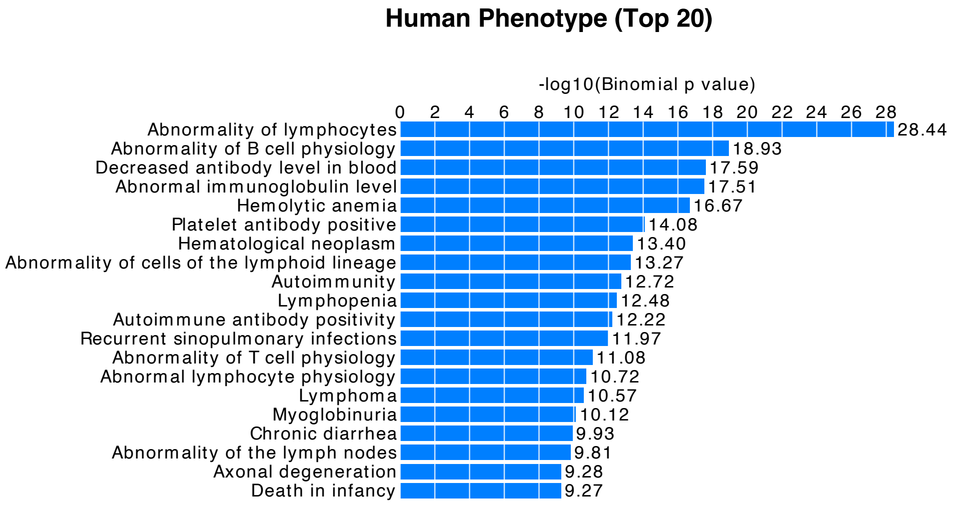


B.

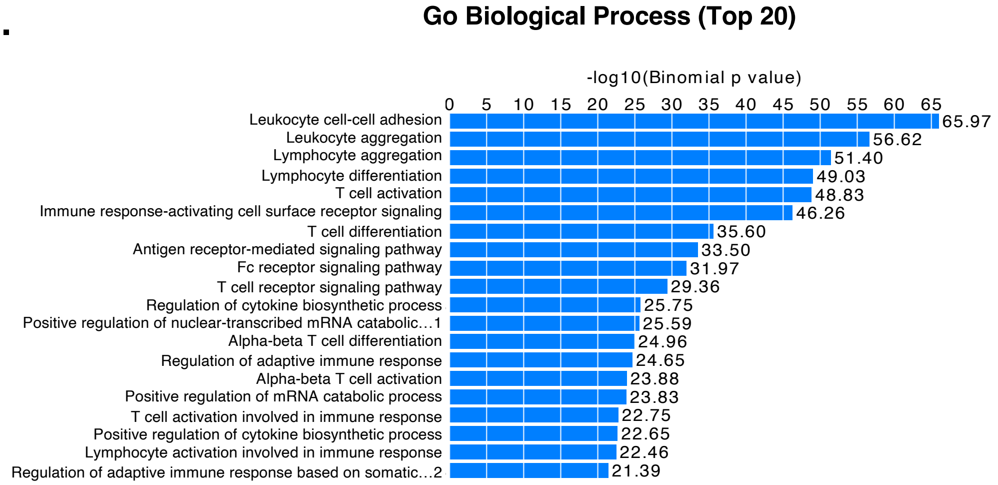


Full name of description
1. positive regulation of nuclear-transcribed mRNA catabolic process, deadenylation-dependent decay
2. regulation of adaptive immune response based on somatic recombination of immune receptors built from Ig superfamily domains

**Supplemental Figure 5. Enrichment analysis for the Human phenotype ontologies and GO Biological Process for patient A.II.1**

GREAT (v.4.04) analysis was performed to identify putative cis-regulatory regions that overlapped the differentially accessible ATAC-seq peaks (adjusted p-value cutoff < 0.05) and to identify enrichment for the GO Biological Process or Human Phenotype ontologies analyses based on genes around the putative cis-regulatory elements. Note: This figure is based on differentially accessible ATAC-seq peaks analysis for Q402* vs. healthy controls; the confidence of this differential analysis is limited due to the lack of an available replicate for the Q402* factor.

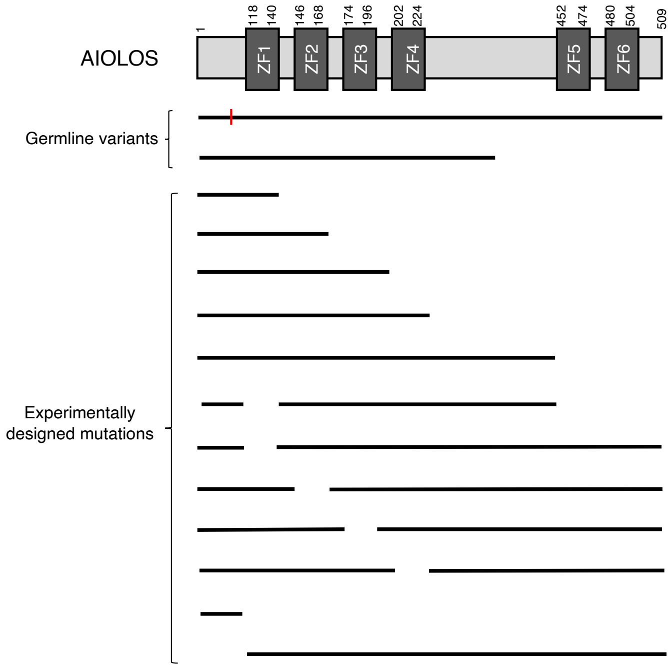


| AIO mutants | Dimerization with AIOLOS WT | Dimerization with the same mutant | Dimerization with IKAROS or HELIOS | PC-HC localization | DNA binding | Reduced protein stability | Ubiquitination | Sumoylation |
|---|---|---|---|---|---|---|---|---|
| E82K | + | | + | + | + | + | + | + |
| Q402* | + | + | - | - | + | + | - | - |
| ZF1 | + | + | | - | - | | | |
| ZF1-2 | + | + | | - | - | | | |
| ZF1-3 | + | + | | - | - | | | |
| ZF1-4 | + | + | | - | + | | | |
| Del ZF5-6 | + | + | | - | + | | | |
| Del ZF1 and ZF5-6 | + | - | | | | | | |
| Del ZF1 | | | | + | + | | | |
| Del ZF2 | | | | - | - | | | |
| Del ZF3 | | | | - | - | | | |
| Del ZF4 | | | | - | - | | | |
| ZF less | | | | | | + | | |
| Del N-term | | | | | | + | | |

**Supplemental Figure 6. Summary of AIOLOS mutants' results**

A schematic diagram of AIOLOS mutants is depicted on the left. The symbol '+' indicates that the effect was observed, and '-' means the variants had no effect on the tests. Gray shading denotes that the mutation was not included in the test. In the DNA binding, Q402*, ZF1-4, and Del ZF5-6 exhibit an abnormal binding pattern, predominantly monomers.

| ID (age and sex) [Reference ranges, % (ALC)] | **A.II.1 (Index) (16y, F)** | **A.II.2 (sister) (9y, F)** | **A.I.1 (Father) (41y, M)** |
|---|---|---|---|
| CD3+ | 80.4 % (***856***) [58-85 % (1000-4900)] | 72.5 % (2595) [66-76 % (1000-5300)] | 83.9 % (2490) [58-85 % (700-3000)] |
| CD3+HLADR+ | 16.7 % (178) [5-15 % (20-400)] | 10.6 % (379) [5-15 % (20-400)] | **16.1** % (**478**) [5-15 % (30-400)] |
| CD3+CD4+ | ***24.5*** % (***261***) [28-64 % (400-2100)] | 33.1 % (1243) [30-65 % (500-2400)] | 30.2 % (897) [30-56 % (400-1400)] |
| CD3+CD8+ | **51.9** % (553) [15-40 % (200-1600)] | 32.8 % (1102) [15-40 % (300-1800)] | **53.4** % (**1585**) [18-45 % (200-1100)] |
| CD4/CD8 ratio | ***0.47*** [0.8-3.5] | 1.01 [0.8-2.5] | ***0.57*** [0.8-2.5] |
| CD3+CD4+CD45RA+ (Naïve CD4 T cells) | ***6.6*** % (***56***) [25-55 % (250-1500)] | ***17.2*** % (446) [30-55 % (300-1900)] | ***6.4*** % (159) [10-35 % (70-850)] |
| CD3+CD8+CD45RA+ (Naïve CD8 T cells) | **37** % (***39***) [18-35 % (180-1400)] | ***10.4*** % (269) [18-40 % (180-1600)] | 28.4 % (**707**) [13-30 % (90-700)] |
| CD3+CD4+CD45RO+- (Memory CD4 T cells) | 22.3 % (191) [15-35 % (150-800)] | 31.3 % (812) [13-35 % (130-1100)] | 30.2 % (752) [30-60 % (200-1000)] |
| CD3+CD8+CD45RO+- (Memory CD8 T cells) | **29.3** % (251) [5-20 % (50-500)] | **32.2** % (**836**) [5-18 % (50-720)] | 33.6 % (**837**) [10-35 % (70-650)] |
| CD4+CD45RA+CD31+ (Recent thymic emigrants) | ***10*** % (***26***) [35-60 % (170-1200)] | ***28.2*** % (333) [40-70 % (200-1600)] | ***11.7*** % (105) [15-40 % (60-600)] |
| CD4+CD25+CD127low (Treg) | 4.2 % (***11***) [3-15 % (12-320)] | 5.8 % (69) [3-15 % (15-350)] | 4.1 % (37) [3-10 % (12-150)] |
| CD16+CD56+ | 6.6 % (***70***) [5-25 % (90-1000)] | 11.7 % (419) [4-24 % (70-900)] | 7.8 % (232) [5-25 % (90-600)] |
| CD3+CD16+CD56+ | **6.3** % (67) [0.1-5 % (2-100)] | 0.3 % (11) [0.1-5 % (2-100)] | **9.8** % (**291**) [0.1-5 % (2-100)] |
| CD19+ | 11.8 % (***126***) [10-22 % (200-600)] | 14.3 % (512) [13-25 % (300-1100)] | 7.5 % (223) [7-20 % (200-500)] |
| CD19+CD27-IgD+ (Naïve B-cells, % of B cells) | **83.2** % (***105***) [65-78 % (130-470)] | **85.6** % (438) [69-80 % (200-900)] | **75.2** % (168) [58-72 % (110-360)] |
| CD19+CD27+IgD- (Switched memory B cells, % of B) | ***3.4*** % (***4***) [7-20 % (14-120)] | ***3.8*** % (19) [4-10 % (12-110)] | 12.6 % (28) [10-20 % (20-150)] |
| CD19+CD27+IgD+ (non-switched memory B cells, % of B) | ***3.0*** % (***4***) [7-15 % (14-100)] | ***2.7*** % (***14***) [5-10 % (15-110)] | ***7.5*** % (***17***) [13-21 % (26-110)] |
| CD19+CD21-CD38- (% of B cells) | 3.6 % (5) [0-5 % (0-30)] | 1.1 % (6) [0-5 % (0-50)] | 4.8 % (11) [0-5 % (0-25)] |
| Neutrophil | 2584 [1800-6500] | 2290 [1800-6500] | n.d |
| Monocyte | 190 [80-800] | 530 [80-800] | n.d |
| Eosinophil | 38 [20-500] | 50 [20-500] | n.d |
| Basophil | 38 [0-100] | 50 [0-100] | n.d |
| IgG | ***4.93*** g/L [5.49-15.84] | ***6.08*** g/L [6.98-15.6] | 10.73 g/L [7-16] |
| IgM | 0.95 g/L [0.23-2.59] | 0.51 g/L [0.31-1.79] | 0.93 g/L [0.4-2.3] |
| IgA | 0.74 g/L [0.61-3.48] | 1.73 g/L [0.53-2.04] | 3.78 g/L [0.7-4] |
| IgE | <0.130 [0-100 IU/ml] | 1.61 [0-100 IU/ml] | 3.91 [0-100 IU/ml] |

| ID (age and sex) | **B.I.1 (72y, F) (Index)** | **B.II.1 (43y, F)** | **B.II.2 (39y, M)** | **C.I.1 (73y, F)** | **C.II.1 (46y, F) (Index)** | **C.III.1 (13y, F)** |
|---|---|---|---|---|---|---|
| CD3+ | **90.2** % (1236)<br>[55-88 % (651-2804)] | **89.8** % (1679)<br>[55-88 % (651-2804)] | 86.6 % (788)<br>[55-88 % (651-2804)] | 62.4 % (***636***)<br>[55-88 % (651-2804)] | 64.2 % (***520***)<br>[55-88 % (651-2804)] | 72.8 % (1441)<br>[65-87 % (1054-3177)] |
| CD3+CD4+ | **71.7** % (982)<br>[28-56 % (370-1336)] | 50.6 % (946)<br>[28-56 % (370-1336)] | 45.4 % (413)<br>[28-56 % (370-1336)] | 42.4 % (432)<br>[28-56 % (370-1336)] | 37.6 % (***305***)<br>[28-56 % (370-1336)] | 45.4 % (899)<br>[31-53 % (605-1665)] |
| CD3+CD8+ | 16.2 % (222)<br>[14-46 % (185-1024)] | 34.9 % (653)<br>[14-46 % (185-1024)] | 33 % (300)<br>[14-46 % (185-1024)] | 19 % (194)<br>[14-46 % (185-1024)] | 22.9 % (185)<br>[14-46 % (185-1024)] | 22.8 % (451)<br>[19-40 % (274-1386)] |
| CD4/CD8 ratio | **4.43**<br>[0.8-4] | 1.45<br>[0.8-4] | 1.38<br>[0.8-4] | 2.23<br>[0.8-4] | 1.64<br>[0.8-4] | 2<br>[0.8-2.8] |
| CD3+CD4+CD45RA+CD62L+<br>(Naïve cells, of CD4) | 56.7%<br>[14-67 %] | 47.8%<br>[14-67 %] | 31.8%<br>[14-67 %] | 44.9%<br>[14-67 %] | 19.6%<br>[14-67 %] | 53.6%<br>[35-76 %] |
| CD3+CD4+CD45RA-CD62L+<br>(Central memory, of CD4) | 33.3%<br>[26-64 %] | 35.5%<br>[26-64 %] | 45.8%<br>[26-64 %] | 30.4%<br>[26-64 %] | 48.9%<br>[26-64 %] | 27.6%<br>[11-41 %] |
| CD3+CD4+CD45RA-CD62L-<br>(Effector memory, of CD4) | 8.7%<br>[4.5-30 %] | 14.3%<br>[4.5-30 %] | 20.8%<br>[4.5-30 %] | 21.2%<br>[4.5-30 %] | 29.8%<br>[4.5-30 %] | 17.2%<br>[6.1-26 %] |
| CD3+CD4+CD45RA+CD62L-<br>(Terminal effector memory, of CD4) | 1.2 %<br>[0-3.7 %] | 2.4 %<br>[0-3.7 %] | 1.6 %<br>[0-3.7 %] | 3.5 %<br>[0-3.7 %] | 1.6 %<br>[0-3.7 %] | 1.7 %<br>[0.5-13 %] |
| CD3+CD8+CD45RA+CD62L+<br>(Naïve cells, of CD8) | 29.1%<br>[25-73%] | 43.9%<br>[25-73%] | 30.2%<br>[25-73%] | ***22***%<br>[25-73%] | 45.5%<br>[25-73%] | 58.1%<br>[33-79 %] |
| CD3+CD8+CD45RA-CD62L+<br>(Central memory, of CD8) | 34.3%<br>[5.9-40 %] | 6.3%<br>[5.9-40 %] | 10.9 %<br>[5.9-40 %] | ***5.1*** %<br>[5.9-40 %] | ***5.2*** %<br>[5.9-40 %] | 3.6 %<br>[0.9-12 %] |
| CD3+CD8+CD45RA-CD62L-<br>(Effector memory, of CD8) | 5.5%<br>[5.5-34%] | 10.2%<br>[5.5-34%] | 15.6 %<br>[5.5-34%] | 23.7 %<br>[5.5-34%] | 12.7 %<br>[5.5-34%] | ***9.1*** %<br>[3.7-24 %] |
| CD3+CD8+CD45RA+CD62L-<br>(Terminal effector memory, of CD8) | 31.1%<br>[4.8-33%] | **39.6**%<br>[4.8-33%] | **43.4** %<br>[4.8-33%] | **49.2** %<br>[4.8-33%] | **36.6** %<br>[4.8-33%] | 29.3 %<br>[10-47 %] |
| CD4+CD45RA+CD31+<br>(Recent thymic emigrants, of CD4) | 31.1%<br>[6.7-45 %] | 34%<br>[6.7-45 %] | 18.3%<br>[6.7-45 %] | 23.2%<br>[6.7-45 %] | 12.4%<br>[6.7-45 %] | 46.3%<br>[28-61 %] |
| CD4+CD25+FOXP3+<br>(Treg, % of CD4) | ***3.2*** %<br>[4-10 %] | 5.3 %<br>[4-10 %] | 5.2 %<br>[4-10 %] | 5.1 %<br>[4-10 %] | 6.3 %<br>[4-10 %] | 2.4 %<br>[2.4-8] |
| CD16+CD56+ (NK) | ***1.4*** % (***19***)<br>[7-33 % (126-841)] | ***6*** % (***112***)<br>[7-33 % (126-841)] | 11.8 % (***107***)<br>[7-33 % (126-841)] | **33.7** % (344)<br>[7-33 % (126-841)] | 30 % (243)<br>[7-33 % (126-841)] | 15.8 % (313)<br>[3.5-22 % (67-456)] |
| CD3+CD16+CD56+ (NKT) | 2.6 % (***36***)<br>[2.1-18 % (56-448)] | 9.6 % (180)<br>[2.1-18 % (56-448)] | 14 % (127)<br>[2.1-18 % (56-448)] | 5.3 % (54)<br>[2.1-18 % (56-448)] | 9.1 % (74)<br>[2.1-18 % (56-448)] | 4.4 % (87)<br>[2.2-9.6 % (42-230)] |
| CD19+ (or CD20+) | 8.6 % (118)<br>[3.8-18 % (79-399)] | 4.3 % (80)<br>[3.8-18 % (79-399)] | ***1.6*** % (***15***)<br>[3.8-18 % (79-399)] | 4.3 % (***44***)<br>[3.8-18 % (79-399)] | 6 % (***49***)<br>[3.8-18 % (79-399)] | 11.2 % (222)<br>[5.4-25 % (130-887)] |
| CD20+CD27-IgM+<br>(Naïve B-cells, of CD20) | **98.5** %<br>[56-92 %] | 84.5 %<br>[56-92 %] | 70.8 %<br>[56-92 %] | 86.8 %<br>[56-92 %] | 81.4 %<br>[56-92 %] | 92 %<br>[74-95 %] |
| CD20+CD27+IgM-<br>(Switched memory B cells, of CD20) | ***0.4*** %<br>[4.3-20 %] | ***4.2*** %<br>[4.3-20 %] | ***3.5*** %<br>[4.3-20 %] | ***4.1*** %<br>[4.3-20 %] | 4.4 %<br>[4.3-20 %] | 3 %<br>[4.3-20 %] |
| CD20+CD27+IgM+<br>(non-switched memory B, of CD20) | ***0.6*** %<br>[4.8-39 %] | 5.5 %<br>[4.8-39 %] | 19.2 %<br>[4.8-39 %] | 6.1 %<br>[4.8-39 %] | 9.4 %<br>[4.8-39 %] | 2.8 %<br>[2.9-13 %] |
| CD19+CD21-CD38- (of CD19) | 0.35 %<br>[0.3-2.2 %] | 0.19 %<br>[0.3-2.2 %] | **11.1** %<br>[0.3-2.2 %] | 0.6 %<br>[0.3-2.2 %] | 0.7 %<br>[0.3-2.2 %] | 0.6 %<br>[0.2-2.8 %] |
| Neutrophil | 2310<br>[1560-6130] | 5170<br>[1560-6130] | 3360<br>[1560-6130] | n.d. | 2790<br>[1560-6130] | n.d. |
| Monocyte | 710<br>[240-860] | 740<br>[240-860] | 720<br>[240-860 | n.d. | 270<br>[240-860 | n.d. |
| Eosinophil | 150<br>[40-360] | 170<br>[40-360] | 230<br>[40-360] | n.d. | 20<br>[40-360] | n.d. |
| Basophil | 60<br>[10-80] | 60<br>[10-80] | 40<br>[10-80] | n.d. | 30<br>[10-80] | n.d. |
| IgG (mg/dL)<br>[Reference] | ***572***, 1262*<br>[600-1540] | 1184<br>[540-1822] | 783<br>[540-1822] | 792<br>[540-1822] | 681<br>[610-1616] | 1182<br>[658-1534] |
| IgM (mg/dL)<br>[Reference] | ***28, 26***<br>[33-293] | 166<br>[22-293] | 60<br>[22-293] | 92<br>[22-293] | **338**<br>[22-293] | 102<br>[48-186] |
| IgA (mg/dL)<br>[Reference] | n.d., 78<br>[68-517] | 128<br>[63-484] | ***34***<br>[63-484] | 105<br>[63-484] | 112<br>[63-484] | 102<br>[47-221] |
| IgE (IU/mL)<br>[Reference] | <25<br>[0-100] | <25<br>[0-100] | <25<br>[0-100] | <25<br>[0-100] | 10<br>[ <=114] | 152<br>[<450] |

**Supplemental Table 1. Laboratory results of immunological tests in AIOLOS haploinsufficiency patients**

Bold indicates above normal ranges, and bold/italic indicates below normal ranges, according to age-matched reference ranges. Reference values are from Belarusian Research Center (Family A) and NIH clinical center (Family B and C). Normal ranges are in square brackets. B.I.1's Ig levels determined in 2013 and 2022. *Patient under IgG replacement therapy. n.d. indicates not determined. ALC indicates absolute lymphocyte number.

| AIOLOS variants | G159R | N160S | Q402* | E82K |
|---|---|---|---|---|
| Mechanism of action | Dominant negative on IKAROS and AIOLOS WT | Dominant negative on AIOLOS WT | Haploinsufficiency (Dimerization defect) | Haploinsufficiency (Low protein stability) |
| Immunological phenotype | | | | |
| Recent thymic emigrants (CD4 T) | n.d. | Increased (3/4) | Decreased (3/3) | Normal (5/6) |
| B cell number | Low (3/3) | Low-high (1 low, 2 normal, 1 high) | Low-normal (1 low, 2 normal) | Low-normal (3 low, 3 normal) |
| Switched memory B | n.d. | Low (4/4) | Low (2/3) | Low (4/6) |
| IgG level | Low (1/3) | Low (4/4) | Low (2/3) | Low (1/6) |
| Clinical phenotype | | | | |
| Bacterial infection | Yes (2/3) | Yes (4/4) | Yes (2/3) | Yes (4/6) |
| *Pneumocystis jirovecii* pneumonia | No | Yes (3/4) | No | No |
| EBV-associated severe diseases | Yes (3/3) | No | No | No |
| Hematological malignancy | B lymphoma (2/3) | CLL (1/4) | No | No |
| Immune dysregulation | No | No | Yes (2/3) ITP, Allergy | Yes (6/6) SLE Hashimoto's thyroiditis, AI hepatitis, Sjogren's-like sicca syndrome, atopy and allergies, ANA positive, non-infectious colitis, polymyalgia rheumatica, recurrent urticaria |

**Supplemental Table 5. Immunological and clinical phenotypes of germline heterozygous IKZF3 mutations in AIOLOS-associated diseases.**

| **Primer sequences used for the plasmid preparation** | |
|---|---|
| Mutagenesis primers | |
| IKZF3 c.244 G>A, p.E82K-Forward | 5’-AACCCATGGGAAATGCAGAAAAGCCTGAAATCCCTTACAGC-3’ |
| IKZF3 c.244 G>A, p.E82K- Reverse | 5’-GCTGTAAGGGATTTCAGGCTTTTCTGCATTTCCCATGGGTT-3’ |
| IKZF3 c.1204C>T, p.Q402*-Forward | 5’-AACGCCAGAATCACATCTATTAGCAAAATCACATGGTCCTG-3’ |
| IKZF3 c.1204C>T, p.Q402*- Reverse | 5’-CAGGACCATGTGATTTTGCTAATAGATGTGATTCTGGCGTT-3’ |
| IKZF3 c.421_423delinsTAG, p.T141*-Forward | 5’-TGGTTCATAAGCGAAGCCATTAGGGTGAACGCCCATTCCAGTG-3’ |
| IKZF3 c.421_423delinsTAG, p.T141*- Reverse | 5’-CACTGGAATGGGCGTTCACCCTAATGGCTTCGCTTATGAACCA-3’ |
| IKZF3 c.505_506delinsTA, p.T169*-Forward | 5’-TCCGCCACATTAAACTGCACTAAGGGGAAAAACCTTTTAAGTG-3’ |
| IKZF3 c.505_506delinsTA, p.T169*- Reverse | 5’-CACTTAAAAGGTTTTTCCCCTTAGTGCAGTTTAATGTGGCGGA-3’ |
| IKZF3 c.590_591delinsAG, p.S197*-Forward | 5’-CGGGGCATCTTAGGACACATTAGGTGGAGAAACCCTACAAATG-3’ |
| IKZF3 c.590_591delinsAG, p.S197*- Reverse | 5’-CATTTGTAGGGTTTCTCCACCTAATGTGTCCTAAGATGCCCCG-3’ |
| IKZF3 c.673_675delinsTGA, p.R225*-Forward | 5’-AGGAGCACAAGGAGCGCTGCTGAACATTTCTTCAGAGCACTGA-3’ |
| IKZF3 c.673_675delinsTGA, p.R225*- Reverse | 5’-TCAGTGCTCTGAAGAAATGTTCAGCAGCGCTCCTTGTGCTCCT-3’ |
| IKZF3 c.1351_1352delinsTA, p.V451*-Forward | 5’-AGGAAGGGGAGGTGATGGATTAGTATCGGTGTGACCACTGCC-3’ |
| IKZF3 c.1351_1352delinsTA, p.V451*- Reverse | 5’-GGCAGTGGTCACACCGATACTAATCCATCACCTCCCCTTCCT-3’ |
| IKZF3 c.352_353delinsGC, p.M118A-Forward | 5’-GCAGGCCAACCAGTGGAAAGGCGAACTGCGATGTGTGTGGAT-3’ |
| IKZF3 c.352_353delinsGC, p.M118A- Reverse | 5’-ATCCACACACATCGCAGTTCGCCTTTCCACTGGTTGGCCTGC-3’ |
| IKZF3 c.355_356delinsGC, p.N119A-Forward | 5’-GGCCAACCAGTGGAAAGATGGCCTGCGATGTGTGTGGATTAT-3’ |
| IKZF3 c.355_356delinsGC, p.N119A- Reverse | 5’-ATAATCCACACACATCGCAGGCCATCTTTCCACTGGTTGGCC-3’ |
| IKZF3 c.365T>C, p.V122A-Forward | 5’-TGGAAAGATGAACTGCGATGCGTGTGGATTATCCTGCATCA-3’ |
| IKZF3 c.365T>C, p.V122A- Reverse | 5’-TGATGCAGGATAATCCACACGCATCGCAGTTCATCTTTCCA-3’ |
| IKZF3 c.373_374delinsGC, p.L125A-Forward | 5’-TGAACTGCGATGTGTGTGGAGCATCCTGCATCAGCTTCAATG-3’ |
| IKZF3 c.373_374delinsGC, p.L125A- Reverse | 5’-CATTGAAGCTGATGCAGGATGCTCCACACACATCGCAGTTCA-3’ |
| IKZF3 c.376T>G, p.S126A-Forward | 5’-ACTGCGATGTGTGTGGATTAGCCTGCATCAGCTTCAATGTC-3’ |
| IKZF3 c.376T>G, p.S126A- Reverse | 5’-GACATTGAAGCTGATGCAGGCTAATCCACACACATCGCAGT-3’ |
| IKZF3 c.385_386delinsGC, p.S129A-Forward | 5’-TGTGTGGATTATCCTGCATCGCCTTCAATGTCTTAATGGTTC-3’ |
| IKZF3 c.385_386delinsGC, p.S129A- Reverse | 5’-GAACCATTAAGACATTGAAGGCGATGCAGGATAATCCACACA-3’ |
| IKZF3 c.388_389delinsGC, p.F130A-Forward | 5’-GTGGATTATCCTGCATCAGCGCCAATGTCTTAATGGTTCATA-3’ |
| IKZF3 c.388_389delinsGC, p.F130A- Reverse | 5’-TATGAACCATTAAGACATTGGCGCTGATGCAGGATAATCCAC-3’ |
| IKZF3 c.349A>T, p.K117*-Forward | 5’-GTAGCAGGCCAACCAGTGGATAGATGAACTGCGATGTGTGT-3’ |
| IKZF3 c.349A>T, p.K117*- Reverse | 5’-ACACACATCGCAGTTCATCTATCCACTGGTTGGCCTGCTAC-3’ |
| Cloning primers | |
| FlagCMV2-AIOLOS-KpnI-Forward | 5’-TCGGTACCAATGGAAGATATACAAACAAAT-3’ |
| FlagCMV2-AIOLOS-XbaI-Reverse | 5’-CTCTAGATCACTTCAGCAGGGCTCTGTG-3’ |
| pcDNA3-HA-AIOLOS-EcoRI- Forward | 5’-GGAATTCATGGAAGATATACAAACAAAT-3’ |
| pcDNA3-HA-AIOLOS-XhoI-Reverse | 5’-CCTCGAGTCACTTCAGCAGGGCTCTGTG-3’ |

| | |
|---|---|
| pcDNA3-HA-AIOLOS (118-509)-EcoRI- Forward | 5’-GGAATTCATGAACTGCGATGTGTG-3’ |
| AIOLOS Del ZF1- Forward | 5’-CCAACCAGTGGAAAGACTGGTGAACGCCCA-3’ |
| AIOLOS Del ZF1-Reverse | 5’-TGGGCGTTCACCAGTCTTTCCACTGGTTGG-3’ |
| AIOLOS Del ZF2- Forward | 5’-ACTGGTGAACGCCCAACAGGGGAAAAACCT-3’ |
| AIOLOS Del ZF2- Reverse | 5’-AGGTTTTTCCCCTGTTGGGCGTTCACCAGT-3’ |
| AIOLOS Del ZF3- Forward | 5’-ACAGGGGAAAAACCTTCTGTGGAGAAACCC-3’ |
| AIOLOS Del ZF3-R Reverse | 5’-GGGTTTCTCCACAGAAGGTTTTTCCCCTGT-3’ |
| AIOLOS Del ZF4- Forward | 5’-TCTGTGGAGAAACCCCGTACATTTCTTCAG-3’ |
| AIOLOS Del ZF4- Reverse | 5’-CTGAAGAAATGTACGGGGTTTCTCCACAGA-3’ |

**Supplemental Table 6. Primer sequences used to generate AIOLOS mutant constructs for this study**
